# Activation of Notch3 in osteoblasts/osteocytes causes compartment-specific changes in bone remodeling

**DOI:** 10.1016/j.jbc.2021.100583

**Published:** 2021-03-24

**Authors:** Ernesto Canalis, Stefano Zanotti, Lauren Schilling, Tabitha Eller, Jungeun Yu

**Affiliations:** 1 Department of Orthopaedic Surgery, UConn Health, Farmington, Connecticut, USA; 2 Department of Medicine, UConn Health, Farmington, Connecticut, USA; 3 UConn Musculoskeletal Institute, UConn Health, Farmington, Connecticut, USA

**Keywords:** osteocyte, osteoblast, Notch receptor, bone, osteoclast, cortical porosity, α-MEM, α-minimum essential medium, BGLAP, bone gamma-carboxyglutamate protein, BMM, bone marrow–derived macrophage, Dmp1, dentin matrix protein 1, FBS, fetal bovine serum, HA, hydroxyapatite, Hes, Hairy Enhancer of Split, Hey, Hes-related with YRPW motif, μCT, microcomputed tomography, MMRC, Mutant Mouse Resource and Research Center, NRR, negative regulatory region, NICD, Notch intracellular domain, qRT-PCR, quantitative RT-PCR, RANKL, receptor activator of nuclear factor-κB, TRAP, tartrate-resistant acid phosphatase

## Abstract

Notch receptors maintain skeletal homeostasis. NOTCH1 and 2 have been studied for their effects on bone remodeling. Although NOTCH3 plays a significant role in vascular physiology, knowledge about its function in other cellular environments, including bone, is limited. The present study was conducted to establish the function of NOTCH3 in skeletal cells using models of *Notch3* misexpression. Microcomputed tomography demonstrated that *Notch3* null mice did not have appreciable bone phenotypes. To study the effects of the NOTCH3 activation in the osteoblast lineage, *BGLAP-Cre* or *Dmp1-Cre* transgenics were crossed with *Rosa*^*Notch3*^ mice, where the NOTCH3 intracellular domain is expressed following the removal of a *loxP*-flanked STOP cassette. Microcomputed tomography demonstrated that *BGLAP-Cre;Rosa*^*Notch3*^ and *Dmp1-Cre;Rosa*^*Notch3*^ mice of both sexes exhibited an increase in trabecular bone and in connectivity, with a decrease in cortical bone and increased cortical porosity. Histological analysis revealed a decrease in osteoclast number and bone resorption in trabecular bone and an increase in osteoclast number and void or pore area in cortical bone of *Rosa*^*Notch3*^ mice. Bone formation was either decreased or could not be determined in *Cre;Rosa*^*Notch3*^ mice. NOTCH3 activation in osteoblasts inhibited *Alpl* (alkaline phosphatase) and *Bglap* (osteocalcin) and induced *Tnfsf11* (RANKL) and *Tnfrsf11b* (osteoprotegerin) mRNA, possibly explaining the trabecular bone phenotype. However, NOTCH3 induced *Tnfsf11* and suppressed *Tnfrsf11b* in osteocytes, possibly explaining the cortical porosity. In conclusion, basal NOTCH3 is dispensable for skeletal homeostasis, whereas activation of NOTCH3 in osteoblasts/osteocytes inhibits osteoclastogenesis and bone resorption in cancellous bone but increases intracortical remodeling and causes cortical porosity.

Notch receptors (NOTCH1 to 4) determine cell fate and function in a variety of cell lineages including those present in bone tissue (1, 2, 3, 4). Consequently, Notch receptors have emerged as major contributors to the regulation of skeletal development and homeostasis. Interactions of specific regions of the extracellular domain of Notch with ligands of the Jagged and Delta-like families lead to the exposure of the negative regulatory region (NRR) and its cleavage by ADAM metalloproteases and the γ-secretase complex (5, 6). The proteolytic cleavage results in the release of the Notch intracellular domain (NICD) and Notch activation (7). The NICD translocates to the nucleus where it forms a complex with recombination signal-binding protein for Ig of κ (RBPJκ) and mastermind-like (MAML) (8, 9, 10). Canonical Notch activation results in the transcription of members of the Hairy Enhancer of Split (*Hes*) and Hes-related with YRPW motif (*Hey*) family of transcription factors (11, 12). Although Notch receptors are activated following interactions with their ligands, a degree of basal activation is possible, particularly in the case of NOTCH3 (13).

Although the four Notch receptors share structural and functional properties, each receptor retains its own identity. This is in part because of their preferential expression in certain cellular environments, interactions with specific Notch ligands, and various degrees of basal activation (3). *Notch1*, *Notch2*, and *Notch3* and low levels of *Notch4* mRNA are expressed by skeletal cells (3, 14, 15). *Notch1*, *Notch2*, and *Notch3* are expressed in cells of the osteoblast lineage, but only *Notch1* and *Notch2* are present in cells of the myeloid/osteoclast lineage, where they have direct effects on osteoclast differentiation (3, 16, 17). NOTCH1 inhibits osteoclast differentiation, whereas NOTCH2 induces osteoclastogenesis by direct actions on the osteoclast lineage and by inducing receptor activator of nuclear factor-κB (NF-κB) ligand (RANKL) in the osteoblast lineage (14, 16, 17, 18). *Notch3* is not expressed in the myeloid lineage, but it has the potential to induce osteoclastogenesis by enhancing the production of RANKL in osteoblasts/osteocytes (16). Notch receptors inhibit osteoblast differentiation (3). Although NOTCH1 and NOTCH2 have been studied extensively for their effects on bone remodeling, less is known regarding the role of NOTCH3 in skeletal physiology.

Recently, we created a mouse model harboring a *Notch3* mutation designed to reproduce the functional outcome of lateral meningocele syndrome and termed *Notch3*^*em1Ecan*^ (synonym *Notch3*^*tm1.1Ecan*^) (16, 19). *Notch3*^*em1Ecan*^ mice exhibit a modest NOTCH3 gain of function, osteopenia, and high bone remodeling indicating that NOTCH3 plays a role in skeletal metabolism. However, to establish the function of NOTCH3 in bone, it is important to define the consequences of wildtype NOTCH3 activation on the skeleton and to determine the skeletal phenotype of mice harboring a *Notch3* inactivation.

In the present work, we attempted to define the role of NOTCH3 by establishing the consequences of its misexpression on bone remodeling. For this purpose, we obtained *Notch3* null and *Rosa*^*Notch3*^ mice, where a *loxP*-flanked STOP cassette was cloned into the *Rosa26* locus upstream of sequences coding for the *Notch3* NICD (20). Because *Notch3* is expressed by osteoblasts/osteocytes, *Rosa*^*Notch3*^ mice were crossed with transgenics expressing Cre under the control of the bone gamma-carboxyglutamate protein (*BGLAP*, osteocalcin) or the dentin matrix protein 1 (*Dmp1*) promoter (21, 22). The phenotype of mice misexpressing NOTCH3 was established by microcomputed tomography (μCT) and histomorphometry and possible mechanisms involved were explored.

## Results

### General characteristics of *Notch3* null mice

**Figure 1:**
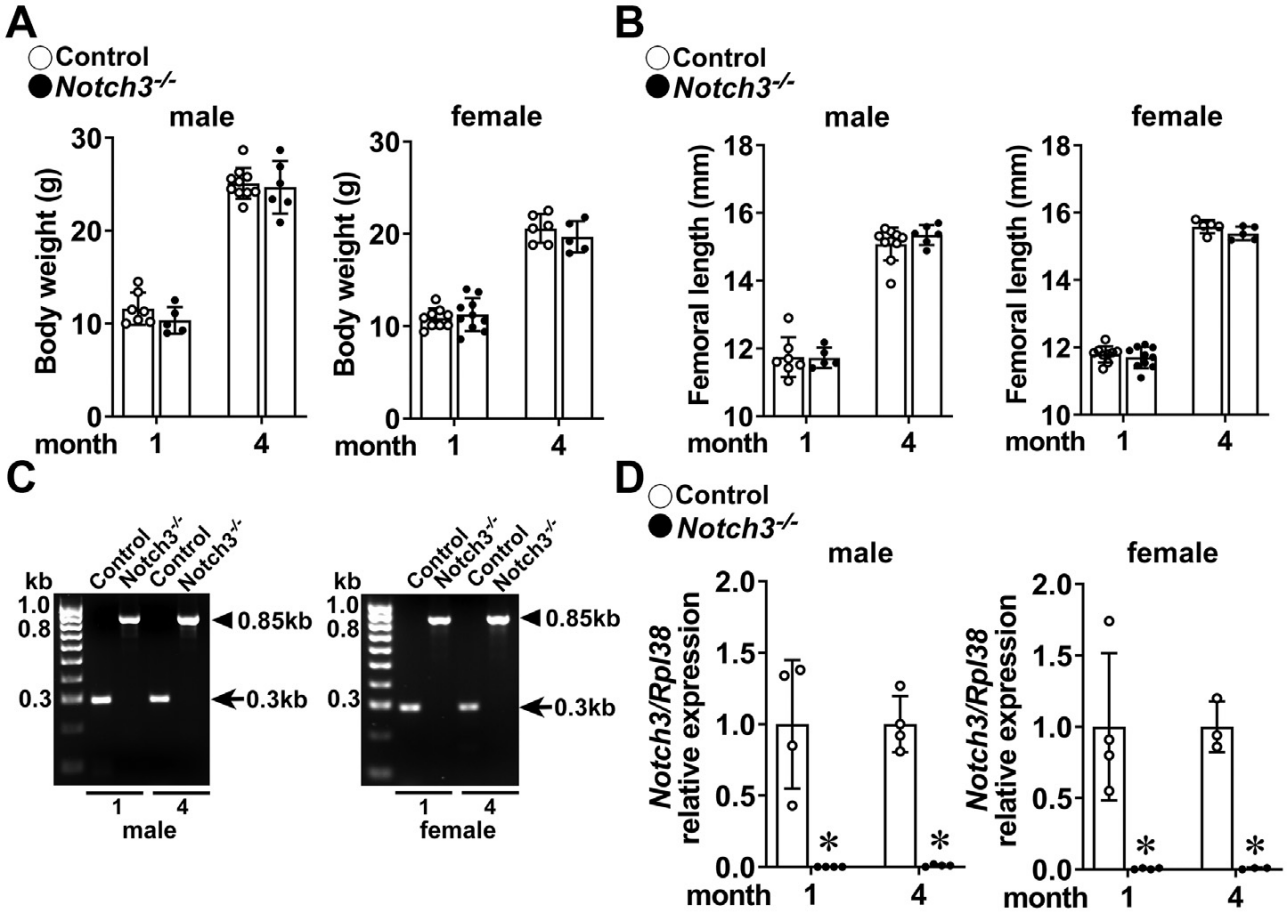
**Weight, femoral length, and identification of *Notch3* null alleles by genotyping of tail DNA and mRNA levels in bone extracts.***A*, body weight and (*B*) femoral length of 1- and 4-month-old *Notch3*^*−/−*^ (*closed circles*) and sex-matched controls (open circles). Bars represent means and ranges SD; n = 7–9 control and n = 5–10 *Notch3*^*−/−*^ at 1 month of age, and n = 6–10 control and n = 6 *Notch3*^*−/−*^ at 4 months of age. *C*, tail DNA was obtained from *Notch3*^*−/−*^ and control mice for genotyping, and (*D*) total RNA was obtained from tibiae from *Notch3*^*−/−*^ (*closed circles*) and sex- and age-matched control (*open circles*) mice. ∗Significantly different between *Notch3*^*−/−*^ and controls by unpaired *t* test.

To study the consequences of the inactivation of *Notch3* in the skeleton, we obtained global *Notch3* null mice from the Mutant Mouse Resource and Research Centers (MMRC) and examined their phenotype following intercrosses of heterozygous *Notch3*^*−/+*^ mice. Although the intent was to compare *Notch3*^*−/−*^ with littermate sex-matched wildtype controls, the yield of the expected genotype (25%) and sex-matching made littermate comparisons not always possible, so that it was necessary to compare mice from different but related litters. *Notch3*^*−/−*^ mice had a healthy appearance, and their weight and femoral length were comparable with those of control mice (Fig. 1). *Notch3* mRNA levels in tibiae from *Notch3*^*−/−*^ mice were undetectable.

### Skeletal microarchitecture of *Notch3* null mice

**Figure 2:**
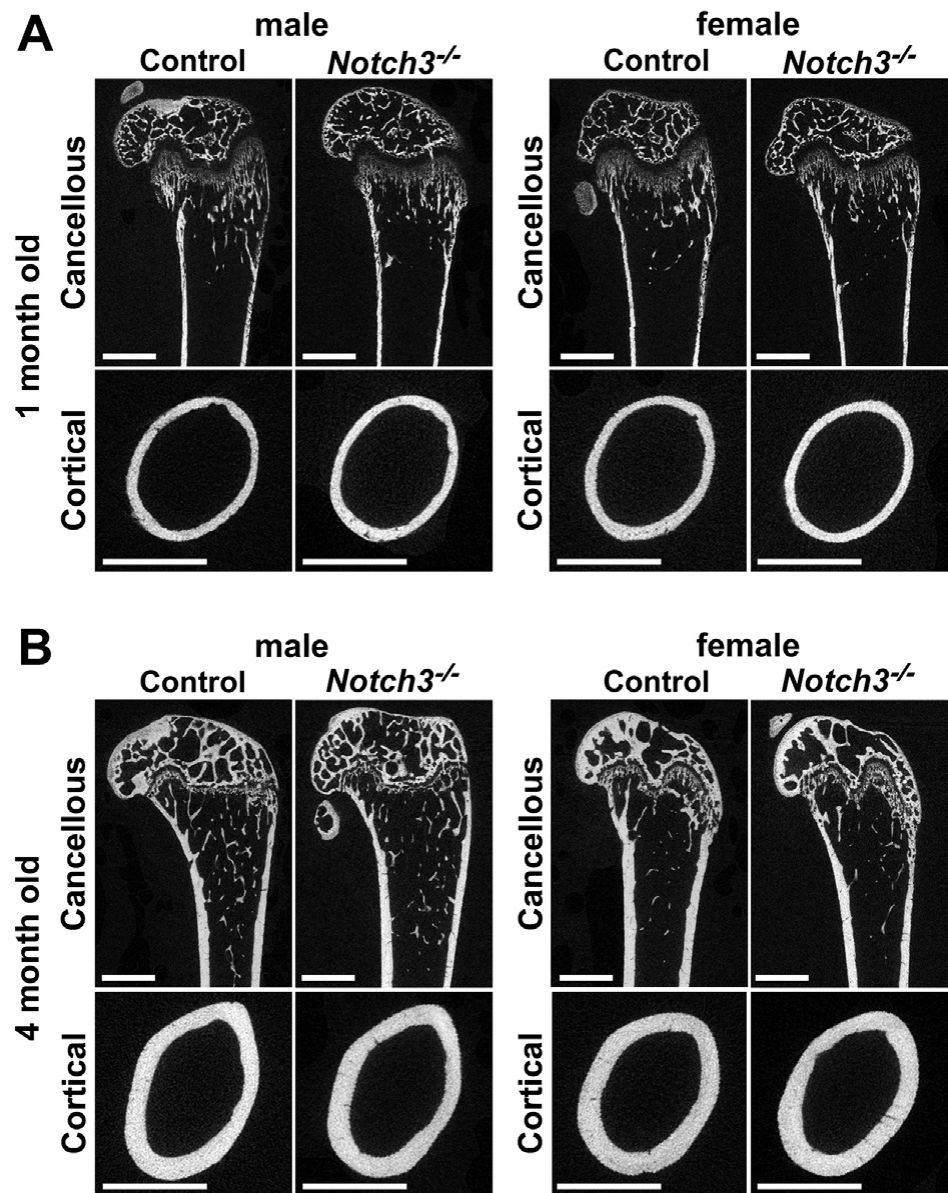
**Representative microcomputed tomography of femurs from (*A*) 1- and (*B*) 4-month-old male and female control and *Notch3***^*−/−*^**mice.** A sagittal cut is shown for cancellous bone, and a cross-sectional cut at mid-diaphysis is shown for cortical bone. Bar in left lower corner scale 1 mm.

Femoral microarchitecture, determined by μCT, of 1- and 4-month-old male *Notch3*^*−/−*^ mice revealed no differences in trabecular or cortical bone when compared with control sex-matched mice of the same age (Table 1, Fig. 2). μCT of 1-month-old female mice revealed no alterations in either cancellous or cortical bone when compared with control mice (Table 1). Femoral bone microarchitecture of 4-month-old female *Notch3*^*−/−*^ mice demonstrated a modest decrease in trabecular thickness and density of material in cancellous bone and a mild decrease in cortical bone thickness and area.

**Table 1:** Femoral microarchitecture assessed by microcomputed tomography of 1- and 4-month-old *Notch3* null male and female mice and sex- and age-matched controls

Males	1 Month		4 Months	
	Control	*Notch3*^*−/−*^	Control	*Notch3*^*−/−*^
Distal femur trabecular bone	n = 6	n = 5	n = 9	n = 5
Bone volume/total volume (%)	7.1 ± 1.5	6.9 ± 0.7	9.1 ± 2.8	8.9 ± 0.8
Trabecular separation (μm)	250 ± 21	258 ± 28	222 ± 11	216 ± 16
Trabecular number (1/mm)	4.1 ± 0.3	3.9 ± 0.3	4.5 ± 0.2	4.6 ± 0.3
Trabecular thickness (μm)	31 ± 2	31 ± 1	39 ± 6	37 ± 2
Connectivity density (1/mm^3^)	208 ± 27	209 ± 17	159 ± 20	184 ± 33
Structure model index	2.4 ± 0.1	2.4 ± 0.1	2.2 ± 0.4	2.2 ± 0.1
Density of material (mg HA/cm^3^)	759 ± 26	747 ± 32	871 ± 13	856 ± 22
Femoral midshaft cortical bone	n = 7	n = 5	n = 10	n = 6
Bone volume/total volume (%)	97.0 ± 1.7	95.8 ± 4.6	99.9 ± 0.1	99.9 ± 0.0
Porosity (%)	3.0 ± 4.7	4.2 ± 4.6	0.1 ± 0.16	0.1 ± 0.0
Cortical thickness (μm)	77 ± 3	71 ± 5	171 ± 10	167 ± 7
Total area (mm^2^)	1.4 ± 0.1	1.3 ± 0.2	1.7 ± 0.2	1.6 ± 0.1
Bone area (mm^2^)	0.3 ± 0.1	0.3 ± 0.1	0.7 ± 0.1	0.7 ± 0.1
Periosteal perimeter (mm)	4.1 ± 0.1	4.0 ± 0.3	4.6 ± 0.2	4.5 ± 0.1
Endocortical perimeter (mm)	3.6 ± 0.1	3.5 ± 0.2	3.5 ± 0.2	3.4 ± 0.1
Density of material (mg HA/cm^3^)	927 ± 32	905 ± 58	1188 ± 20	1179 ± 40
p Moment of inertia (mm^4^)	0.126 ± 0.02	0.103 ± 0.02	0.321 ± 0.07	0.288 ± 0.04

Females	1 Month		4 Months	
	Control	*Notch3*^*−/−*^	Control	*Notch3*^*−/−*^
Distal femur trabecular bone	n = 9	n = 9	n = 7	n = 5
Bone volume/total volume (%)	6.4 ± 0.9	6.8 ± 1.2	4.1 ± 0.6	3.3 ± 1.0
Trabecular separation (μm)	282 ± 26	268 ± 27	311 ± 34	332 ± 40
Trabecular number (1/mm)	3.6 ± 0.3	3.8 ± 0.4	3.3 ± 0.4	3.0 ± 0.4
Trabecular thickness (μm)	32 ± 1	32 ± 2	35 ± 3	31 ± 1^a^
Connectivity density (1/mm^3^)	179 ± 24	193 ± 44	81 ± 19	76 ± 22
Structure model index	2.3 ± 0.1	2.3 ± 0.2	2.8 ± 0.1	2.8 ± 0.2
Density of material (mg HA/cm^3^)	767 ± 14	767 ± 22	877 ± 14	850 ± 26^a^
Femoral midshaft cortical bone	n = 9	n = 10	n = 7	n = 5
Bone volume/total volume (%)	98.2 ± 0.8	97.9 ± 1.6	99.9 ± 0	99.8 ± 0.1
Porosity (%)	1.8 ± 0.8	2.1 ± 1.6	0.1 ± 0	0.2 ± 0.1
Cortical thickness (μm)	80 ± 6	77 ± 6	180 ± 6	170 ± 5^a^
Total area (mm^2^)	1.4 ± 0.2	1.3 ± 0.1	1.6 ± 0.2	1.6 ± 0.1
Bone area (mm^2^)	0.3 ± 0.1	0.3 ± 0.1	0.74 ± 0.05	0.69 ± 0.02^a^
Periosteal perimeter (mm)	4.2 ± 0.3	4.0 ± 0.1	4.5 ± 0.2	4.4 ± 0.1
Endocortical perimeter (mm)	3.6 ± 0.4	3.5 ± 0.1	3.4 ± 0.3	3.3 ± 0.2
Density of material (mg HA/cm^3^)	932 ± 23	930 ± 38	1206 ± 25	1171 ± 30
p Moment of inertia (mm^4^)	0.119 ± 0.02	0.111 ± 0.02	0.303 ± 0.06	0.270 ± 0.03

Microcomputed tomography was performed in distal femurs for trabecular bone and midshaft for cortical bone from 1- and 4-month-old *Notch3* null mice and age- and sex-matched controls. Values means ± SD.

a Significantly different between control and *Notch3*^*−/−*^, *p* < 0.05 by unpaired *t* test.

### Bone marrow stromal cell cultures from *Notch3* null mice

**Figure 3:**
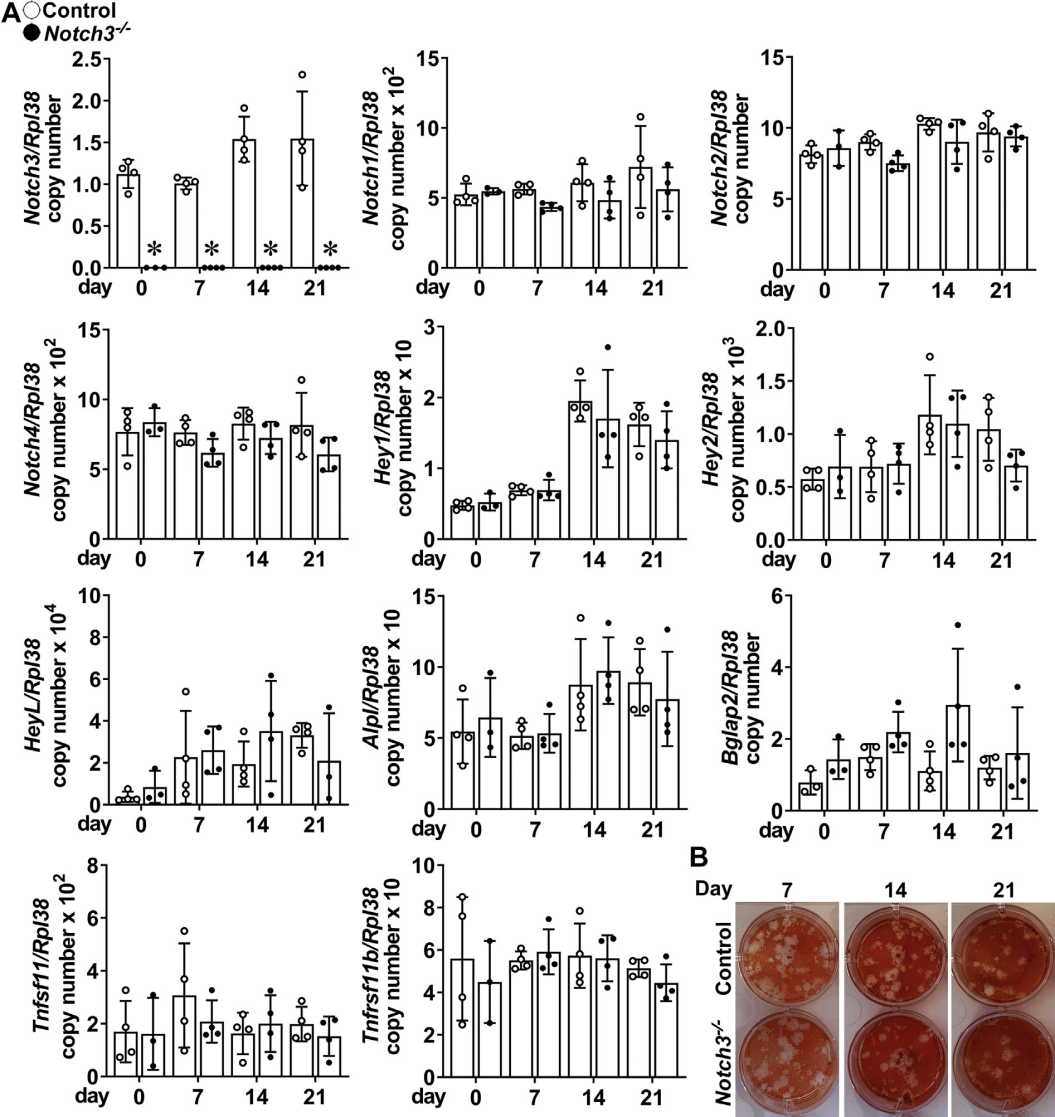
**Bone marrow stromal cells harvested from tibiae of 5-week-old *Notch3***^*−/−*^**(*closed circles*) and control wildtype mice (*open circles*) were cultured for 21 days following confluence (Day 0).***A*, total RNA was extracted and gene expression determined by qRT-PCR. Data are expressed as *Notch3*, *Notch1*, *Notch2*, *Notch4*, *Hey1*, *Hey2*, *HeyL*, *Alpl*, *Bglap*, *Tnfsf11* (RANKL), and *Tnfrsf11b* (osteoprotegerin) copy number corrected for *Rpl38* expression. Bars represent means and ranges SD; n = 4 biological replicates. *Alpl* and *Bglap* in control and experimental cultures were not statistically different between 7, 14, and 21 days and day 0 by two-way ANOVA. ∗Significantly different between *Notch3*^*−/−*^ and controls, *p* < 0.05. *B*, representative alizarin red staining of mineralized nodules in control (*top*) or *Notch3*^*−/−*^ (*bottom*) cultures.

To determine whether NOTCH3 had a direct effect in cells of the osteoblast lineage, bone marrow stromal cells from *Notch3* null and control mice were isolated and cultured to confluence (5–7 days) prior to study (day 0). The inactivation of *Notch3* did not result in appreciable changes in the Notch target genes *Hey1*, *Hey2*, and *HeyL* or in the expression of *Notch1*, *Notch2*, and *Notch4* when compared with control cultures (Fig. 3). mRNA levels of *Alpl* and *Bglap* (osteocalcin) were stable throughout the culture period, and *Alpl*, *Bglap*, *Tnfsf11* (RANKL), and *Tnfrsf11b* (osteoprotegerin) were not significantly different between *Notch3*^*−/−*^ mice and controls. In addition, there was a modest increase in mineralized nodules as control cultures progressed but no difference in the formation of mineralized nodules in cultures from *Notch3* null mice when compared with controls (Fig. 3).

### Osteocyte-enriched cells from *Notch3* null mice

Because *Notch3* is prominently expressed by osteocytes and because these cells play a major role in the regulation of bone remodeling through the RANKL/osteoprotegerin axis, we examined for the expression of Notch target genes and *Tnfsf11* and *Tnfrsf11b* in osteocyte-rich preparations from *Notch3*^*−/−*^ and control mice (15, 23, 24). The inactivation of *Notch3* did not alter the expression of the Notch target genes *Hey1*, *Hey2*, *HeyL*, or *Hes1* and did not have an effect on *Tnfsf11* (RANKL) or *Tnfrsf11b* (osteoprotegerin) mRNA levels compared with control wildtype cells (not shown).

### General characteristics of *Rosa*^*Notch3*^ mice

**Figure 4:**
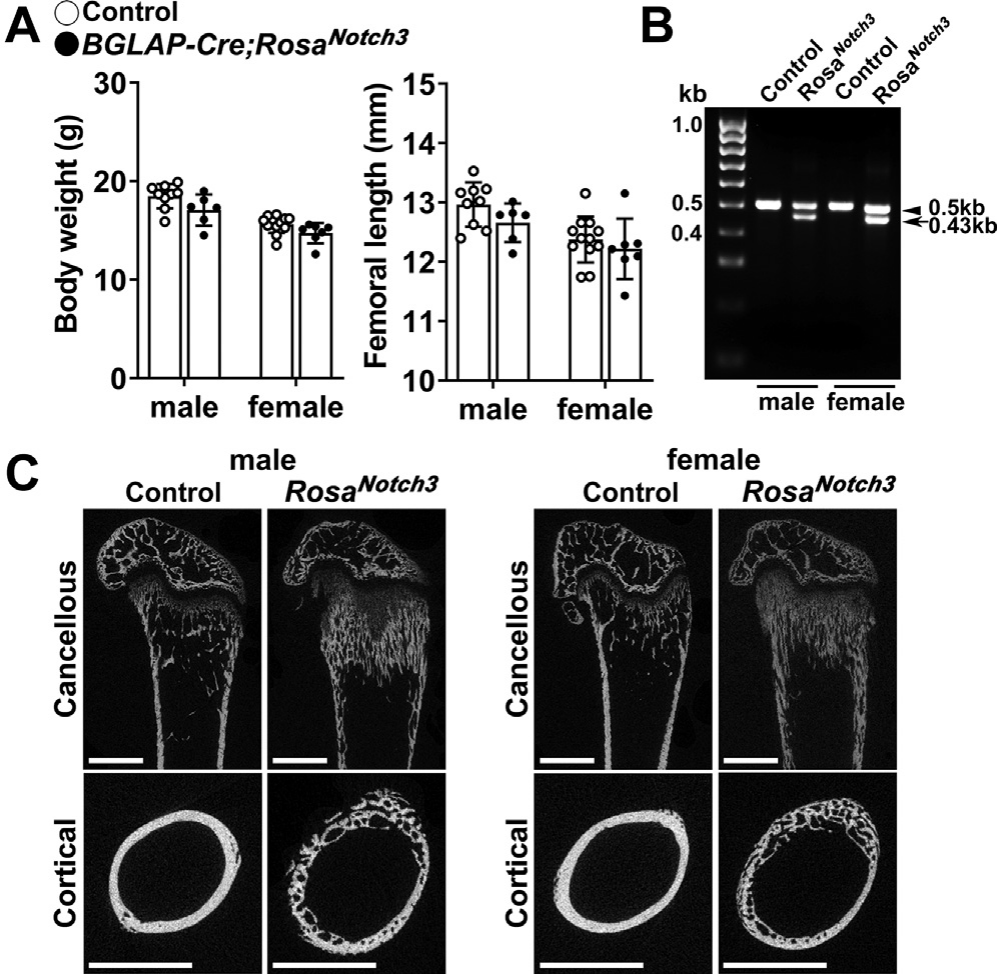
**Weight, femoral length, validation of *loxP* recombination, and representative microcomputed tomography images of 1-month-old male and female *BGLAP-Cre;Rosa***^***Notch3***^**mice.***A*, body weight and femoral length of 1-month-old *BGLAP-Cre;Rosa*^*Notch3*^ (*closed circles*) and sex-matched littermate controls (*open circles*). Bars represent means and ranges SD; n = 9 control males and n = 12 control females and n = 6 (males) and 7 (females) *BGLAP-Cre;Rosa*^*Notch3*^. *B*, DNA extracted from tibiae of *BGLAP-Cre;Rosa*^*Notch3*^ and control mice before and following *loxP* recombination by Cre under the control of the *BGLAP* promoter is shown. A 504-bp band is detected for the allele not recombined, and a 432-bp band is detected for the recombined allele. *C*, representative microcomputed tomography of femurs from 1-month-old male and female control and *BGLAP-Cre;Rosa*^*Notch3*^ mice. A sagittal cut is shown for cancellous bone, and a cross-sectional cut at mid-diaphysis shown for cortical bone. Bar in left lower corner scale 1 mm.

**Figure 5:**
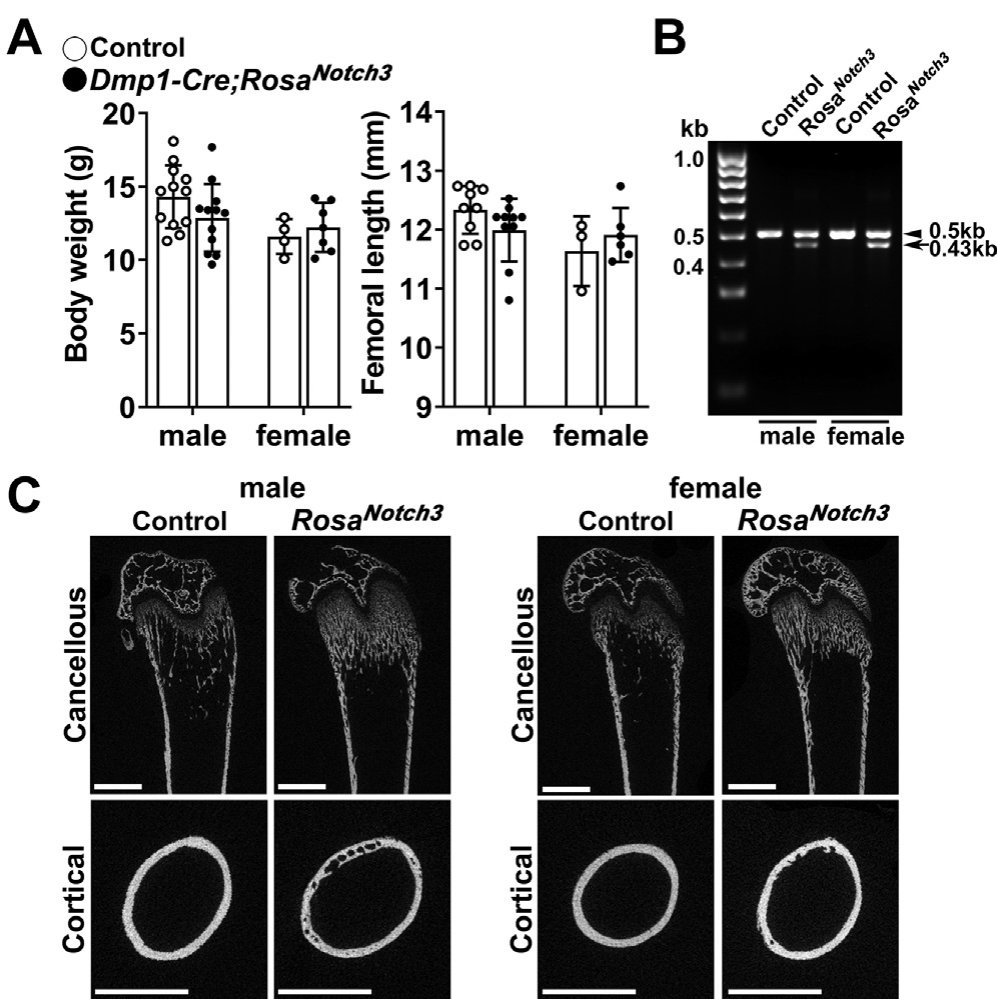
**Weight, femoral length, validation of *loxP* recombination, and representative microcomputed tomography images of 1-month-old male and female *Dmp1-Cre;Rosa***^***Notch3***^**mice.***A*, body weight and femoral length of 1-month-old *Dmp1-Cre;Rosa*^*Notch3*^ (*closed circles*) and sex-matched littermate controls (*open circles*). Bars represent means and ranges SD; n = 3 or 4 (females) and 9 or 12 (males) control and n = 6 or 7 (females) and 10 or 12 (males) *Dmp1-Cre;Rosa*^*Notch3*^. *B*, DNA extracted from tibiae of *Dmp1-Cre;Rosa*^*Notch3*^ and control mice before and following *loxP* recombination by Cre under the control of the *Dmp1* promoter is shown. A 504-bp band is detected for the allele not recombined, and a 432-bp band is detected for the recombined allele. *C*, representative microcomputed tomography of femurs from 1-month-old male and female control and *Dmp1-Cre;Rosa*^*Notch3*^ mice. A sagittal cut is shown for cancellous bone and a cross-sectional cut at mid-diaphysis is shown for cortical bone. Bar in left lower corner scale 1 mm.

To induce the conditional activation of NOTCH3 in cells of the osteoblast lineage, homozygous *Rosa*^*Notch3*^ mice were mated with heterozygous *BGLAP-Cre* or *Dmp1-Cre* mice so that approximately half of the pups would express NOTCH3 NICD (*Cre*^*+*^*;Rosa*^*Notch3*^) and approximately half would serve as controls (*Rosa*^*Notch3*^). Cre-mediated recombination of *loxP* sites flanking the STOP cassette was documented in extracts from tibiae of 1-month-old mice. The general appearance of *BGLAP-Cre;Rosa*^*Notch3*^ or *Dmp1-Cre;Rosa*^*Notch3*^ was normal, and their weight and femoral length were not significantly different from those of control littermates (Figs. 4 and 5).

### Skeletal microarchitecture and histomorphometry of *Rosa*^*Notch3*^ mice

In contrast to the absence of a phenotype in *Notch3*^*−/−*^ mice, the expression of the NOTCH3 NICD in mature osteoblasts or in osteocytes caused a significant 1.7- to 2.4-fold increase in cancellous bone volume/tissue volume in *BGLAP-Cre;Rosa*^*Notch3*^ or a 1.5- to 1.8-fold increase in *Dmp1-Cre;Rosa*^*Notch3*^ mice (Tables 2 and 3, Figs. 4 and 5) The higher trabecular bone volume in *BGLAP-Cre;Rosa*^*Cre*^ and *Dmp1-Cre;Rosa*^*Notch3*^ was mostly observed in the distal metaphysis and was associated with a pronounced increase in connectivity density and a lower structure model idex indicating a tendency toward plate-like trabeculae. The trabecular and cortical bone of the distal metaphyses of *BGLAP-Cre;Rosa*^*Notch3*^ and *Dmp1-Cre;Rosa*^*Notch3*^ mice were not clearly distinguishable, suggesting defective or delayed bone corticalization (25). At mid-diaphysis, cortical bone volume and cortical thickness were decreased and the cortex appeared porous in *BGLAP-Cre;Rosa*^*Notch3*^ and *Dmp1-Cre;Rosa*^*Notch3*^ mice of both sexes (Tables 2 and 3, Figs. 4 and 5). Polar moment of inertia was decreased in *Dmp1-Cre;Rosa*^*Notch3*^ mice, suggesting decreased bone strength, although the effect was significant only in male mice, and it was not observed in *BGLAP-Cre;Rosa*^*Notch3*^ mice (26). There were no major sex differences in the phenotype observed, and skeletal microarchitecture was affected similarly in male and female *BGLAP-Cre;Rosa*^*Notch3*^ and *Dmp1-Cre;Rosa*^*Notch3*^ mice. There was a distortion of the normal skeletal microarchitecture in both cortical and cancellous compartments of experimental mice. However, periosteal perimeter, width, and anteroposterior length of the metaphysis of *BGLAP-Cre;Rosa*^*Notch3*^ and *Dmp1-Cre;Rosa*^*Notch3*^ mice were not different from controls (Tables 2 and 3).

**Table 2:** Femoral microarchitecture and dimensions assessed by microcomputed tomography of 1-month-old *BGLAP-Cre;Rosa*^*Notch3*^ male and female mice and sex-matched littermate controls

Trabecular and cortical bone	Males		Females	
	Control n = 9	*Cre*^*+/−*^*;Rosa*^*Notch3*^ n = 6	Control n = 12	*Cre*^*+/−*^*;Rosa*^*Notch3*^ n = 7
Distal femur trabecular bone				
Bone volume/total volume (%)	13.0 ± 2.6	22.4 ± 3.9^b^	9.7 ± 1.6	23.5 ± 7.2^b^
Trabecular separation (μm)	167 ± 15	146 ± 17^b^	186 ± 18	170 ± 61
Trabecular number (1/mm)	6.0 ± 0.5	7.5 ± 1.0^b^	5.4 ± 0.5	7.3 ± 2.3^b^
Trabecular thickness (μm)	34 ± 3	35 ± 1	31 ± 2	37 ± 2^b^
Connectivity density (1/mm^3^)	369 ± 83	852 ± 180^b^	297 ± 71	942 ± 429^b^
Structure model index	1.9 ± 0.3	0.7 ± 0.3^b^	2.2 ± 0.2	0.4 ± 0.2^b^
Density of material (mg HA/cm^3^)	825 ± 22	862 ± 18^b^	808 ± 28	840 ± 27^b^
Distal femur dimensions				
Periosteal perimeter (mm)	6.3 ± 0.2	6.4 ± 0.3	6.0 ± 0.7	6.3 ± 0.2
Anteroposterior length (mm)	1.8 ± 0.1	1.7 ± 0.1	1.7 ± 0.1	1.7 ± 0.1
Mediolateral width (mm)	2.7 ± 0.1	2.6 ± 0.1	2.7 ± 0.1	2.5 ± 0.1
Femoral midshaft cortical bone				
Bone volume/total volume (%)	98.0 ± 1.3	47.5 ± 4.5^b^	98.8 ± 0.6	53.1 ± 8.2^b^
Porosity (%)	2.0 ± 1.3	52.5 ± 4.5^b^	1.2 ± 0.6	46.9 ± 8.2^b^
Cortical thickness (μm)	95 ± 6	36 ± 3^b^	95 ± 5	36 ± 5^b^
Total area (mm^2^)	1.6 ± 0.1	2.0 ± 0.2^b^	1.5 ± 0.1	1.8 ± 0.2^b^
Bone area (mm^2^)^a^	0.43 ± 0.03	0.37 ± 0.09	0.40 ± 0.03	0.30 ± 0.06^b^
Periosteal perimeter (mm)	4.4 ± 0.1	5.0 ± 0.3^b^	4.3 ± 0.2	4.8 ± 0.2^b^
Endocortical perimeter (mm)	3.7 ± 0.1	4.0 ± 0.2^b^	3.7 ± 0.1	4.0 ± 0.1^b^
Density of material (mg HA/cm^3^)	1018 ± 38	934 ± 11^b^	1011 ± 39	916 ± 43^b^
*p* Moment of inertia (mm^4^)	0.183 ± 0.02	0.182 ± 0.04	0.166 ± 0.025	0.157 ± 0.045

Microcomputed tomography was performed in distal femurs for trabecular bone and midshaft for cortical bone from 1-month-old *BGLAP-Cre;Rosa*^*Notch3*^ mice and sex-matched littermate controls.

a Segmented bone. Values means ± SD.

b Significantly different between control and *Cre*^*+/−*^*;Rosa*^*Notch3*^, *p* < 0.05 by unpaired *t* test.

**Table 3:** Femoral microarchitecture and dimensions assessed by microcomputed tomography of 1-month-old *Dmp1-Cre;Rosa*^*Notch3*^ male and female mice and sex-matched littermate controls

Trabecular and cortical bone	Males		Females	
	Control n = 12	*Cre*^*+/−*^*;Rosa*^*Notch3*^ n = 11	Control n = 4	*Cre*^*+/−*^*;Rosa*^*Notch3*^ n = 7
Distal femur trabecular bone				
Bone volume/total volume (%)	11.3 ± 1.8	17.1 ± 3.4^b^	9.6 ± 0.9	14.4 ± 3.3^b^
Trabecular separation (μm)	170 ± 5	152 ± 13^b^	182 ± 13	162 ± 33
Trabecular number (1/mm)	5.9 ± 0.2	7.0 ± 0.5^b^	5.6 ± 0.3	6.6 ± 1.3
Trabecular thickness (μm)	31 ± 3	31 ± 3	29 ± 1	29 ± 2
Connectivity density (1/mm^3^)	384 ± 52	962 ± 217^b^	395 ± 52	908 ± 341^b^
Structure model index	2.1 ± 0.2	1.4 ± 0.5^b^	2.1 ± 0.1	1.6 ± 0.3^b^
Density of material (mg HA/cm^3^)	785 ± 22	799 ± 22	776 ± 7	768 ± 20
Distal femur dimensions				
Periosteal perimeter (mm)	6.1 ± 0.2	6.1 ± 0.2	5.7 ± 0.4	6.2 ± 0.1
Anteroposterior length (mm)	1.8 ± 0.2	1.7 ± 0.1	1.7 ± 0.1	1.7 ± 0.1
Mediolateral width (mm)	2.5 ± 0.1	2.5 ± 0.1	2.4 ± 0.1	2.4 ± 0.1
Femoral midshaft cortical bone				
Bone volume/total volume (%)	96.8 ± 2.4	85.3 ± 6.5^b^	97.2 ± 1.7	81.3 ± 9.0^b^
Porosity (%)	3.2 ± 2.4	14.7 ± 6.5^b^	2.8 ± 1.7	18.7 ± 9.0^b^
Cortical thickness (μm)	88 ± 8	55 ± 11^b^	80 ± 7	48 ± 8^b^
Total area (mm^2^)	1.4 ± 0.1	1.4 ± 0.1	1.3 ± 0.1	1.4 ± 0.1
Bone area (mm^2^)+	0.38 ± 0.03	0.292 ± 0.06^b^	0.32 ± 0.02	0.26 ± 0.01^b^
Periosteal perimeter (mm)	4.2 ± 0.1	4.2 ± 0.2	4.0 ± 0.1	4.2 ± 0.1
Endocortical perimeter (mm)	3.6 ± 0.1	3.7 ± 0.1	3.4 ± 0.2	3.7 ± 0.1^b^
Density of material (mg HA/cm^3^)	955 ± 32	917 ± 17^b^	927 ± 31	895 ± 19
*p* Moment of inertia (mm^4^)	0.151 ± 0.02	0.120 ± 0.03^b^	0.111 ± 0.01	0.103 ± 0.01

Microcomputed tomography was performed in distal femurs for trabecular bone and midshaft for cortical bone from 1-month-old *Dmp1-Cre;Rosa*^*Notch3*^ mice and sex-matched littermate controls.

a Segmented bone. Values means ± SD.

b Significantly different between control and *Cre*^*+/−*^*;Rosa*^*Notch3*^, *p* < 0.05 by unpaired *t* test.

**Figure 6:**
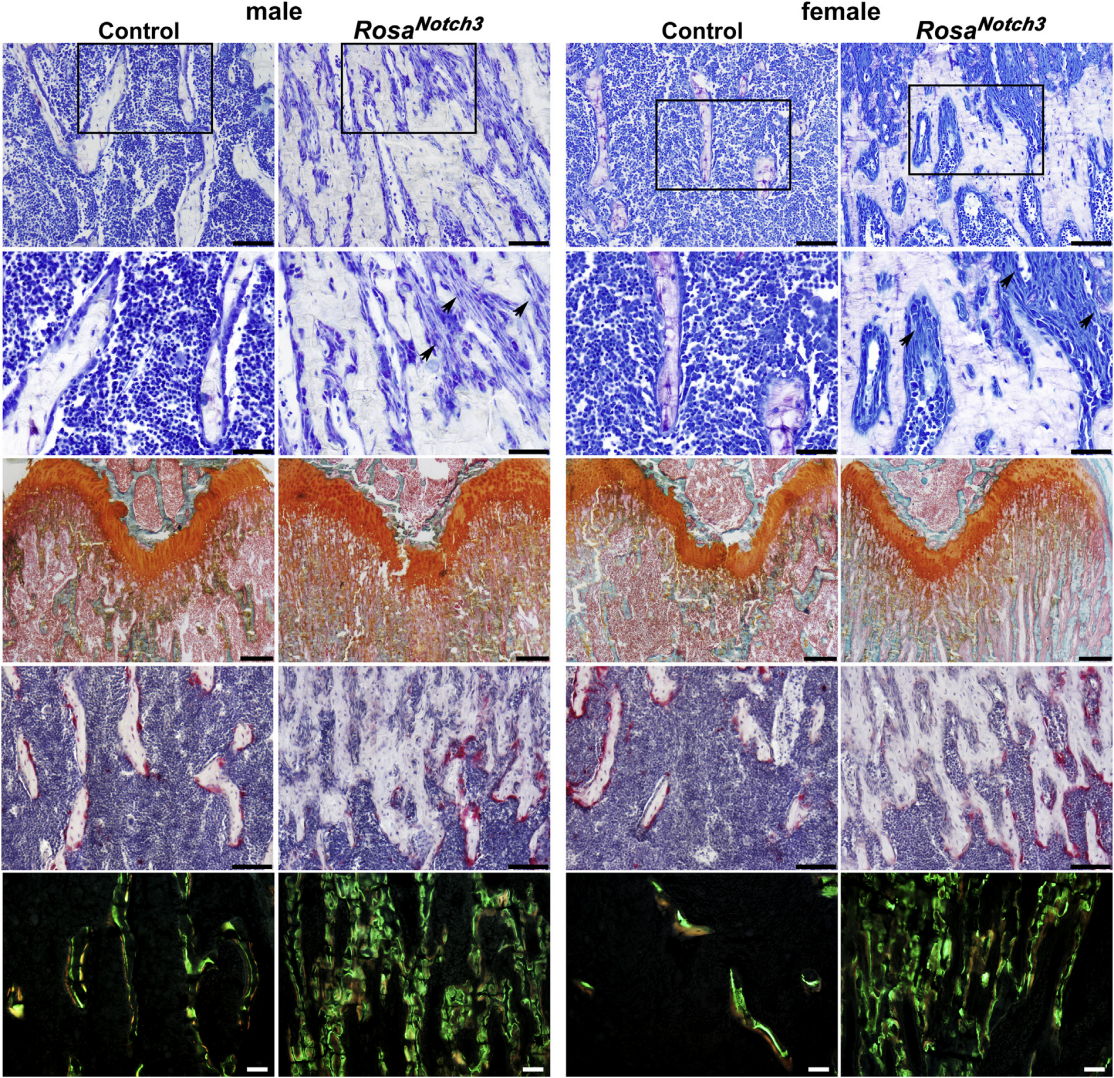
**Representative cancellous bone histomorphometry of femurs from 1-month-old male and female *BGLAP-Cre;Rosa***^***Notch3***^**mice.** Sections embedded in methyl methacrylate were deplasticized and stained with toluidine *blue* (*two upper panels*; scale bars 100 and 50 μm); *arrows* point to fibroblast-like cells or with safranin O and fast *green* (*middle upper panel*; scale bar, 200 μm). Bones embedded in paraffin were processed and stained with TRAP (*red-pink*) and hematoxylin (*blue-purple*) (*fourth panel from top*: scale bar, 100 μm). Calcein (*green*) and demeclocycline (*orange*) labeling of methyl methacrylate embedded sections is shown in the *lower panel* (scale bar, 50 μm).

**Figure 7:**
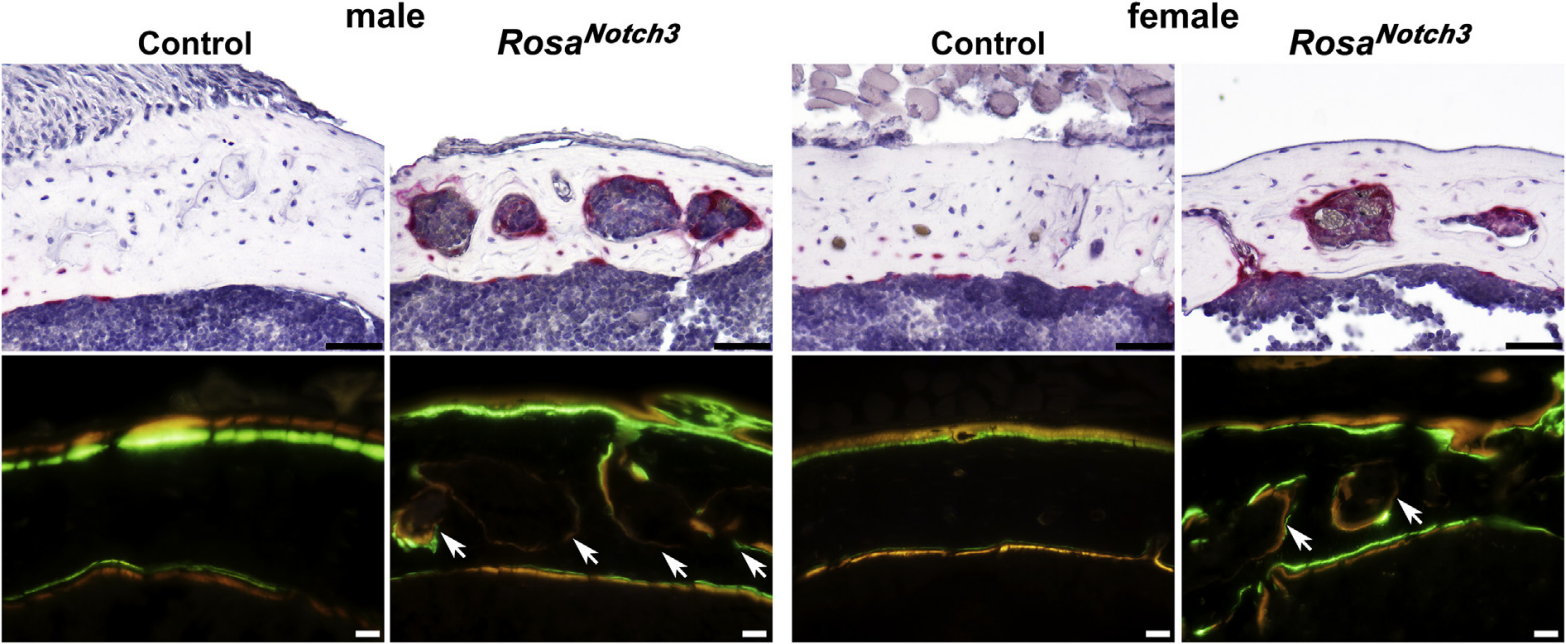
**Representative cortical bone histomorphometry at mid-diaphysis of femurs from 1-month-old male and female *BGLAP-Cre;Rosa***^***Notch3***^**mice.** Bones embedded in paraffin were processed and stained with TRAP (*red-pink*) and hematoxylin (*blue-purple*) (*upper panel*; scale bar, 50 μm). Frozen bones were sectioned to demonstrate calcein (*green*) and demeclocycline (*orange*) labeling (*lower panel*; scale bar, 20 μm). The *arrows* indicate voids or pores in intracortical bone of male and female *BGLAP-Cre;Rosa*^*Notch3*^ mice.

Bone histomorphometry was conducted in male and female mice and sex-matched controls. In accordance with the more pronounced microarchitectural phenotype in *BGLAP-Cre;Rosa*^*Notch3*^ mice, changes in histomorphometric parameters were more evident in this cohort than in *Dmp1-Cre;Rosa*^*Notch3*^ mice. Histomorphometric analysis of cancellous femoral bone (in methyl methacrylate embedded sections) confirmed the increase in bone volume and trabecular number in *BGLAP-Cre;Rosa*^*Notch3*^ mice (Table 4). This was secondary to a decrease in bone resorption since osteoclast number was significantly reduced in *BGLAP-Cre;Rosa*^*Notch3*^ mice and eroded surface was significantly decreased in *BGLAP-Cre;Rosa*^*Notch3*^ male mice (Table 4). Safranin O and fast green staining revealed cartilage remnants in cancellous bone (Fig. 6). Analysis of decalcified bone samples (embedded in paraffin) confirmed a decrease in tartrate-resistant acid phosphatase (TRAP)-positive multinucleated osteoclasts in cancellous bone from male and female *BGLAP-Cre;Rose*^*Notch3*^ mice. The number of osteoclasts/bone perimeter (1/mm) decreased from (means ± SD; n = 4) 5.1 ± 1.2 in control to 2.5 ± 1.6 (*p* < 0.05) in *BGLAP-Cre;Rosa*^*Notch3*^ male mice and from 7.8 ± 2.1 in control to 2.3 ± 1.2 (*p* < 0.05) in *BGLAP-Cre;Rosa*^*Notch3*^ female mice. A decrease in osteoblast number was noted in *BGLAP-Cre;Rosa*^*Notch3*^ mice that reached statistical significance in female mice, and osteoblasts had a fibroblast-like appearance. Calcein/demeclocycline labels did not condense or were missing, not allowing the measurement of mineral apposition rate or mineralizing surface in *BGLAP-Cre;Rosa*^*Notch3*^ male mice (Fig. 6). Bone formation rate was reduced in female mice albeit not significantly. In contrast to the results observed in cancellous bone, cross-sectional analysis of cortical bone revealed an increase in void or pore area and TRAP-positive osteoclast number in male and female *BGLAP-Cre;Rosa*^*Notch3*^ mice demonstrating increased intracortical remodeling. An increase in TRAP-positive osteoclast number also was noted on the endocortical surface of *BGLAP-Cre;Rosa*^*Notch3*^ male mice indicating enhanced resorption in cortical bone contrasting the suppression of bone resorption in cancellous bone (Table 4, Fig. 7).

**Table 4:** Cancellous and cortical bone histomorphometry of 1-month-old *BGLAP-Cre;Rosa*^*Notch3*^ male and female mice and sex-matched *Rosa*^*Notch3*^ control littermates

Cancellous bone	Males		Females	
	Control n = 6	*Cre;Rosa*^*Notch3*^ n = 5	Control n = 6	*Cre;Rosa*^*Notch3*^ n = 4
Static histomorphometry				
Bone volume/tissue volume (%)	17.2 ± 3.8	47.9 ± 11.2^a^	9.5 ± 3.4	42.1 ± 0.9^a^
Trabecular separation (μm)	201 ± 44	39 ± 13^a^	373 ± 139	55 ± 23^a^
Trabecular number (1/mm)	4.3 ± 0.9	13.9 ± 3.1^a^	2.7 ± 0.9	11.6 ± 3.9^a^
Trabecular thickness (μm)	41 ± 7	36 ± 9	35 ± 5	37 ± 4
Osteoblast surface/bone surface (%)	19.1 ± 5.2	14.1 ± 3.6	20.3 ± 3.0	15.9 ± 5.8
Osteoblasts/bone perimeter (1/mm)	15.8 ± 5.5	10.4 ± 2.0	17.4 ± 2.6	11.9 ± 4.1^a^
Osteoid surface/bone surface (%)	0.9 ± 0.9	0.2 ± 0.1	0.9 ± 0.7	0.2 ± 0.2
Osteocytes (mm^2^)	941 ± 118	919 ± 107	1083 ± 200	805 ± 50^a^
Osteoclast surface/bone surface (%)	7.1 ± 1.6	3.5 ± 1.0^a^	9.3 ± 3.3	3.8 ± 1.2^a^
Osteoclasts/bone perimeter (1/mm)	2.9 ± 0.6	1.5 ± 0.4^a^	3.9 ± 1.2	1.6 ± 0.3^a^
Eroded surface/bone surface (%)	2.9 ± 0.8	1.2 ± 0.3^a^	2.1 ± 0.9	1.6 ± 0.4
Dynamic histomorphometry				
Mineral apposition rate (μm/day)	2.1 ± 0.5	ND	1.7 ± 0.3	1.5 ± 0.3
Mineralizing surface/bone surface (%)	1.7 ± 0.7	ND	2.4 ± 1.3	0.8 ± 0.6^b^
Bone formation rate (μm^3^/μm^2^/day)	0.034 ± 0.01	ND	0.041 ± 0.03	0.012 ± 0.01

Cortical bone	Males		Females	
	Control	*Cre;Rosa*^*Notch3*^	Control	*Cre;Rosa*^*Notch3*^
Static histomorphometry	n = 5	n = 5	n = 3	n = 3
Cortical thickness (μm)	127 ± 9	83 ± 14^a^	107 ± 10	84 ± 16
Periosteal perimeter (mm)	5.6 ± 0.6	7.4 ± 1.9	5.5 ± 0.06	6.0 ± 0.87
Endocortical perimeter (mm)	4.8 ± 0.5	8.0 ± 2.1^a^	5.1 ± 0.2	5.9 ± 3.0
Void/pore perimeter (mm)	0.5 ± 0.8	7.3 ± 3.9^a^	0.8 ± 0.7	4.9 ± 2.6^b^
Void/pore area (mm^2^)	0.006 ± 0.009	0.12 ± 0.06^a^	0.004 ± 0.004	0.07 ± 0.06
Void/pore area/bone area (mm^2^)	0.008 ± 0.01	0.22 ± 0.08^a^	0.008 ± 0.007	0.16 ± 0.09^a^
Osteoclasts/bone area (mm^2^)	7.1 ± 10.3	124.6 ± 41.5^a^	8.6 ± 7.4	110 ± 41^a^
Osteocytes/bone area (mm^2^)	1207 ± 59	1207 ± 71	1310 ± 38	1176 ± 24^a^
Endocortical surface				
Static histomorphometry	n = 4	n = 4	n = 3	n = 3
Osteoclast surface/bone surface (%)	7.3 ± 1.7	15.4 ± 4.5^a^	10.6 ± 2.4	15.1 ± 6.2
Osteoclast number/perimeter (1/mm)	4.2 ± 0.5	8.0 ± 1.6^a^	5.5 ± 0.8	7.9 ± 2.7
Eroded surface/bone surface (%)	6.3 ± 3.1	8.6 ± 1.0	6.4 ± 3.0	6.0 ± 1.6
Dynamic histomorphometry	n = 3	n = 3	n = 3	n = 4
Mineral apposition rate (μm/day)	1.15 ± 0.33	0.91 ± 0.29	0.97 ± 0.18	0.87 ± 0.20
Mineralizing surface/bone surface (%)	14.9 ± 8.7	7.9 ± 4.2	9.3 ± 5.2	9.3 ± 5.6
Bone formation rate (μm^3^/μm^2^/day)	0.18 ± 0.14	0.08 ± 0.06	0.08 ± 0.03	0.09 ± 0.06

Histomorphometry was carried out on sagittal sections of distal femurs or TRAP-stained and unstained cross sections at the femoral mid-diaphysis from 1-month-old male and female *BLGAP-Cre;Rosa*^*Notch3*^ mice and sex-matched control littermates. Values are means ± S.D.

a Significantly different between control and *Cre;Rosa*^*Notch3*^*, p* < 0.05 by unpaired *t* test. ND, not determined.

b *p* < 0.055.

Bone histomorphometric analysis of cancellous bone of *Dmp1-Cre;Rosa*^*Notch3*^ male, but not female mice, demonstrated similar, although less pronounced, changes than those observed in *BGLAP-Cre;Rosa*^*Notch3*^ mice (Table 5). Osteoclast number and bone formation were reduced. Analysis of TRAP-positive multinucleated cells in paraffin-embedded sections revealed a nonsignificant ∼20% to 25% decrease in male and female *Dmp1-Cre;Rosa*^*Notch3*^ mice compared with controls (not shown). Cortical histomorphometry could be performed in male *Dmp1-Cre;Rosa*^*Notch3*^ mice only due to the limited number of female mice available for study and revealed a substantial increase in cortical TRAP-positive osteoclasts and void or pore area (over 10-fold), although it did not reach statistical significance owing to variability in the results (Table 5). An increase in osteoclast number was found in the endocortical surface of *Dmp1-Cre;Rosa*^*Notch3*^ male mice.

**Table 5:** Cancellous and cortical bone histomorphometry of 1-month-old *Dmp1-Cre;Rosa*^*Notch3*^ male and female mice and sex-matched *Rosa*^*Notch3*^ control littermates

Cancellous bone	Males		Females	
	Control n = 6	*Cre;Rosa*^*Notch3*^ n = 8	Control n = 3	*Cre;Rosa*^*Notch3*^ n = 7
Static histomorphometry				
Bone volume/tissue volume (%)	14.5 ± 3.2	20.3 ± 5.0^a^	9.0 ± 1.8	9.9 ± 3.9
Trabecular separation (μm)	203 ± 40	122 ± 40^a^	317 ± 31	306 ± 138
Trabecular number (1/mm)	4.3 ± 0.9	7.0 ± 1.8^a^	2.9 ± 0.3	3.4 ± 1.3
Trabecular thickness (μm)	33 ± 8	29 ± 3^a^	31 ± 8	29 ± 3
Osteoblast surface/bone surface (%)	18.2 ± 2.1	20.7 ± 4.6	21.6 ± 5.0	26.0 ± 7.1
Osteoblasts/bone perimeter (1/mm)	15.0 ± 2.0	16.9 ± 4.9	20.2 ± 7.9	23.1 ± 6.6
Osteoid surface/bone surface (%)	0.5 ± 0.7	0.2 ± 0.1^a^	1.4 ± 1.2	0.2 ± 0.2^a^
Osteocytes/bone area (mm^2^)	1042 ± 73	1027 ± 96	1179 ± 150	1117 ± 161
Osteoclast surface/bone surface (%)	8.6 ± 1.3	5.0 ± 0.9^a^	12.2 ± 2.8	10.9 ± 3.9
Osteoclasts/bone perimeter (1/mm)	3.3 ± 0.5	2.1 ± 0.3^a^	5.1 ± 1.3	4.4 ± 1.5
Eroded surface/bone surface (%)	2.9 ± 0.9	2.1 ± 0.5	4.0 ± 1.2	3.8 ± 1.4
Dynamic histomorphometry				
Mineral apposition rate (μm/day)	2.0 ± 0.4	1.7 ± 0.2	1.8 ± 0.4	2.0 ± 0.9
Mineralizing surface/bone surface (%)	1.0 ± 0.3	0.4 ± 0.2^a^	1.9 ± 1.8	0.8 ± 0.6
Bone formation rate (μm^3^/μm^2^/day)	0.022 ± 0.018	0.007 ± 0.003^a^	0.068 ± 0.041	0.017 ± 0.015^a^

Cortical bone	Males	
	Control	*Cre;Rosa*^*Notch3*^
Static histomorphometry	n = 6	n = 5
Cortical thickness (μm)	127 ± 11	103 ± 22
Periosteal perimeter (mm)	5.2 ± 0.7	4.6 ± 0.9
Endocortical perimeter (mm)	4.3 ± 0.7	4.5 ± 1.2
Void/pore perimeter (mm)	0.3 ± 0.4	3.1 ± 3.3
Void/pore area (mm^2^)	0.002 ± 0.002	0.029 ± 0.02
Void/pore area/bone area (mm^2^)	0.003 ± 0.003	0.05 ± 0.04^b^
Osteoclasts/bone area (mm^2^)	1.4 ± 2.0	32.3 ± 32.2
Osteocytes/bone area (mm^2^)	1094 ± 74	1135 ± 181
Endocortical surface		
Static histomorphometry	n = 6	n = 6
Osteoclast surface/bone surface (%)	7.1 ± 3.6	11.2 ± 4.1
Osteoclast number/perimeter (1/mm)	3.5 ± 1.7	6.5 ± 2.7^a^
Eroded surface/bone surface (%)	3.9 ± 2.6	7.2 ± 3.1^a^
Dynamic histomorphometry	n = 5	n = 4
Mineral apposition rate (μm/day)	0.91 ± 0.25	0.58 ± 0.38
Mineralizing surface/bone surface (%)	7.0 ± 4.8	7.6 ± 5.9
Bone formation rate (μm^3^/μm^2^/day)	0.070 ± 0.06	0.058 ± 0.04

Histomorphometry was carried out on sagittal sections of distal femurs or TRAP-stained and unstained cross sections at the femoral mid-diaphysis from 1-month-old *Dmp1-Cre;Rosa*^*Notch3*^ mice and sex-matched control littermates. Values are means ± S.D.

a Significantly different between control and *Cre;Rosa*^*Notch3*^*, p* < 0.05 by unpaired *t* test.

b *p* < 0.055.

### Gene expression profile in bones from *Rosa*^*Notch3*^ mice

**Figure 8:**
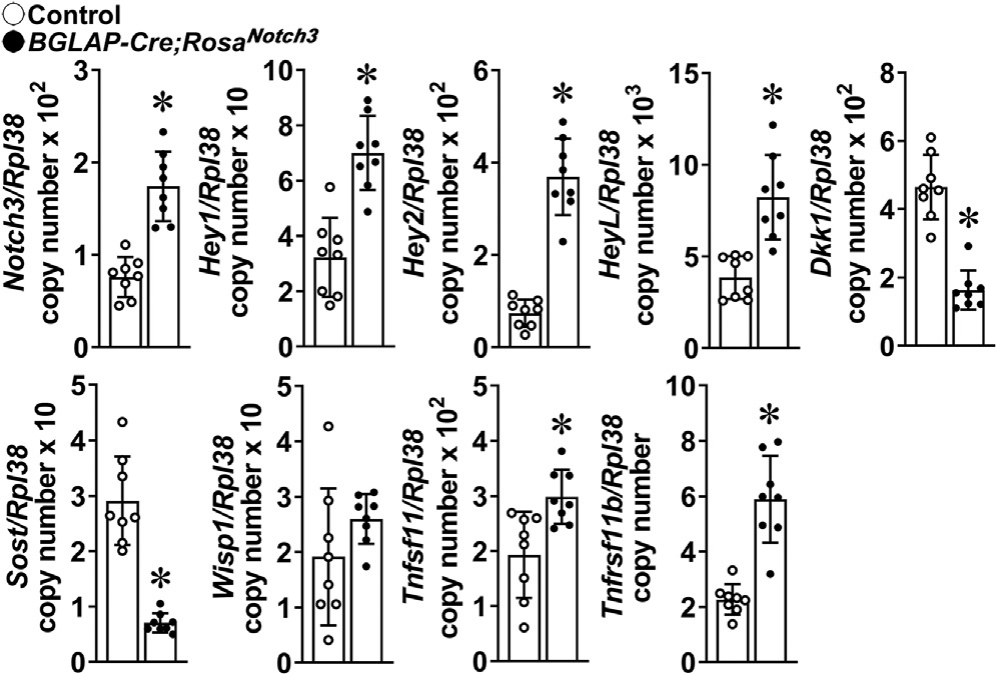
**Total RNA was extracted from tibiae from *BGLAP-Cre;Rosa***^***Notch3***^**(*closed circles*) and age-matched littermate control (*open circles*) mice, and gene expression was determined by qRT-PCR.** Data are expressed as *Notch3NICD, Hey1*, *Hey2*, *HeyL*, *Dkk1*, *Sost*, *Wisp1, Tnfsf11* (RANKL), and *Tnfrsf11b* (osteoprotegerin) copy number corrected for *Rpl38*. Bars represent means and ranges SD; n = 8. Data are derived from biological replicates. ∗Significantly different between *BGLAP-Cre;Rosa*^*Notch3*^ and controls, *p* < 0.05 by unpaired *t* test.

**Figure 9:**
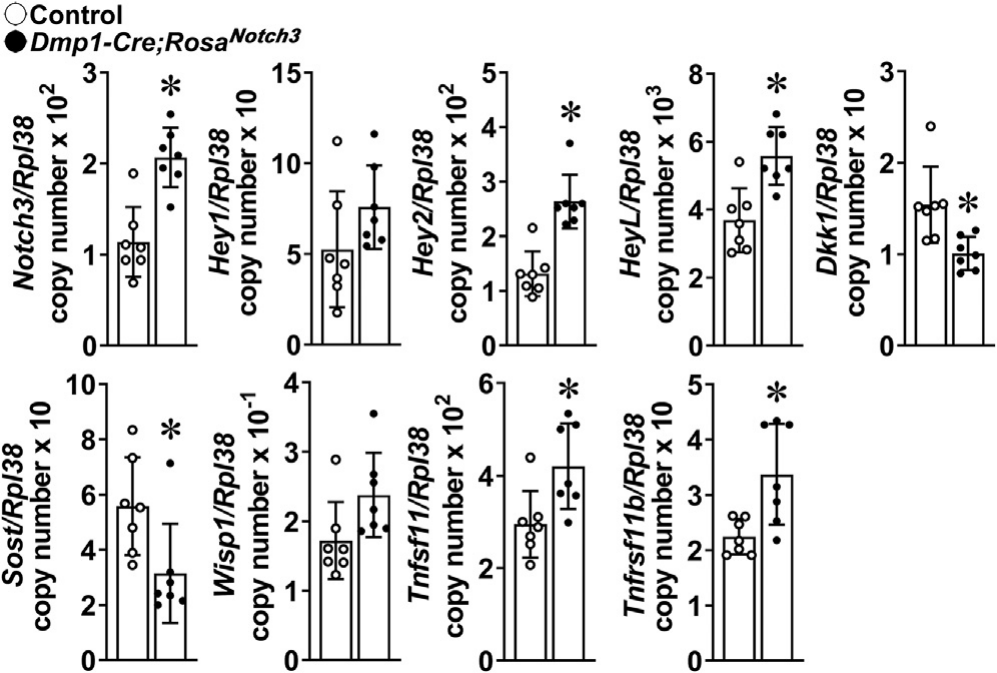
**Total RNA was extracted from tibiae from *Dmp1-Cre;Rosa***^***Notch3***^**(*closed circles*) and age-matched littermate control (*open circles*) mice, and gene expression was determined by qRT-PCR.** Data are expressed as *Notch3NICD, Hey1*, *Hey2*, *HeyL*, *Dkk1*, *Sost*, *Wisp1, Tnfsf11* (RANKL), and *Tnfrsf11b* (osteoprotegerin) copy number corrected for *Rpl38*. Bars represent means and ranges SD; n = 7. Data are derived from biological replicates. ∗Significantly different between *Dmp1-Cre;Rosa*^*Notch3*^ and controls, *p* < 0.05 by unpaired *t* test.

To explore mechanisms that may explain the phenotype of *BGLAP-Cre;Rosa*^*Notch3*^ and *Dmp1-Cre;Rosa*^*Notch3*^ mice, tibiae were analyzed for changes in gene expression. Transcript levels for the *Notch3* NICD and its target genes *Hey1*, *Hey2*, and *HeyL* were increased in tibiae from *BGLAP-Cre;Rosa*^*Notch3*^ and *Dmp1-Cre;Rosa*^*Notch3*^ mice confirming activation of Notch signaling in skeletal cells (Figs. 8 and 9). Notch activation caused an increase in *Tnfrsf11b* (osteoprotegerin) and *Tnfsf11* (RANKL) expression. The induction of *Tnfrsf11b* was more pronounced than that of *Tnfsf11* in tibiae from *BGLAP-Cre;Rosa*^*Notch3*^ mice. *BGLAP-Cre;Rosa*^*Notch3*^ and *Dmp1-Cre;Rosa*^*Notch3*^ mice exhibited a marked suppression of the Wnt antagonists *Sost* (encoding sclerostin) and *Dickkopf 1* (*Dkk1*) mRNA and a modest not statistically significant increase in the Wnt target gene *Wisp1* suggesting enhanced Wnt signaling.

### Bone marrow stromal cell cultures from *Rosa*^*Notch3*^ mice

**Figure 10:**
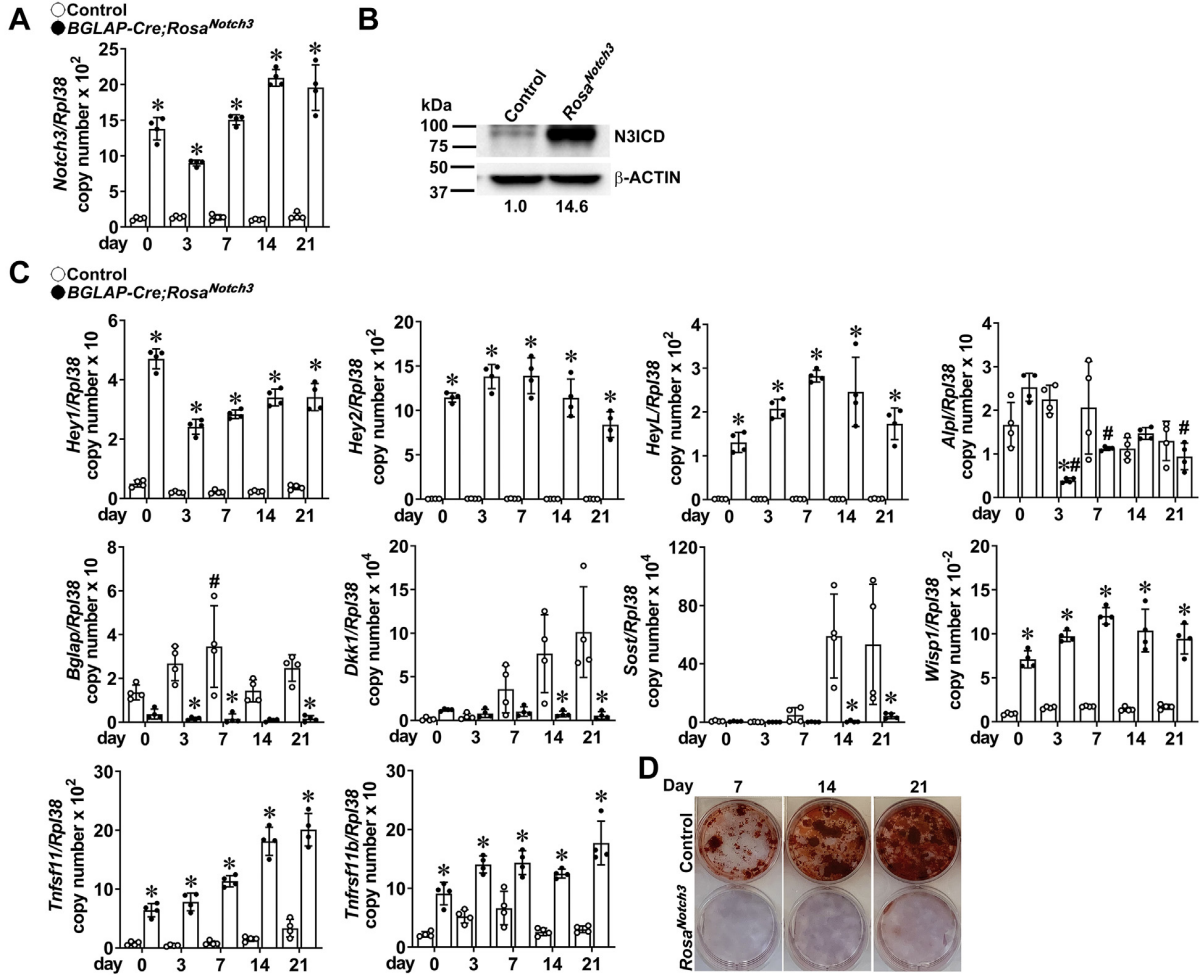
**Bone marrow stromal cells harvested from tibiae of 5-week-old *BGLAP-Cre;Rosa***^***Notch3***^**(*closed circles*) and control littermate mice (*open circles*) were cultured for 21 days following confluence (Day 0).***A* and *C*, total RNA was extracted and gene expression determined by qRT-PCR. Data are expressed as *Notch3-Nicd*, *Hey1*, *Hey2*, *HeyL*, *Alpl*, *Bglap, Dkk1*, *Sost*, *Wisp1*, *Tnfsf11* (RANKL), and *Tnfrsf11b* (osteoprotegerin), copy number corrected for *Rpl38*. Bars represent means and ranges SD; n = 4. Data are derived from technical replicates. ∗Significantly different between *BGLAP-Cre;Rosa*^*Notch3*^ and controls, *p* < 0.05 by two-way ANOVA. #Significantly different from time 0 by two-way ANOVA. *B*, immunoblot demonstrating the presence of NOTCH3 intracellular domain (N3ICD). *D*, representative alizarin red staining of mineralized nodules in control (*top*) or *BGLAP-Cre;Rosa*^*Notch3*^ cultures (*bottom*).

To determine the direct effects of NOTCH3 in cells of the osteoblast lineage, bone marrow stromal cells from *BGLAP-Cre;Rosa*^*Notch3*^ and control mice were isolated and cultured for 21 days. *BGLAP-Cre;Rosa*^*Notch3*^ cells displayed an expected increase in *Notch3-NICD* mRNA and protein by immunoblot, confirming the overexpression of the NICD (Fig. 10). Activation of NOTCH3 resulted in a sustained increase in the Notch target genes *Hey1*, *Hey2*, and *HeyL* as well as an induction of *Tnfsf11* (RANKL) and *Tnfrsf11b* (osteoprotegerin). *Sost* mRNA was expressed at low levels in control cultures and was suppressed further in *BGLAP-Cre;Rosa*^*Notch3*^ cells. *Dkk1* expression was suppressed and the Wnt target gene *Wisp1* was increased suggesting enhanced Wnt signaling. Despite this effect, osteoblastogenesis was suppressed by the activation of NOTCH3 and there was a decrease in mineralized nodule formation and osteocalcin expression in cultures from *BGLAP-Cre;Rosa*^*Notch3*^ mice (Fig. 10). In addition, a transient decrease in *Alp1* was observed.

### Osteocyte-enriched cells from *Rosa*^*Notch3*^ mice

**Figure 11:**
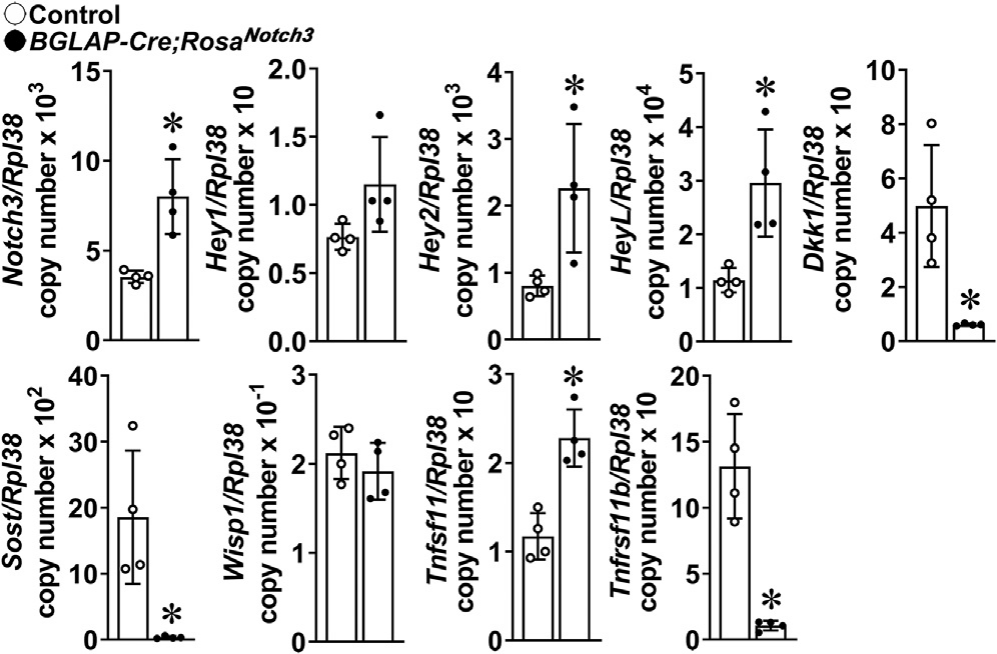
**Total RNA isolated from osteocyte-enriched cells from *BGLAP-Cre;Rosa***^***Notch3***^**(*closed circles*) and control (*open circles*) littermate mice was extracted, and gene expression was determined by qRT-PCR.** Data are expressed as *Notch3-Nicd*, *Hey1*, *Hey2*, *HeyL*, *Dkk1*, *Sost, Wisp1 Tnfsf11* (RANKL), and *Tnfrsf11b* (osteoprotegerin) copy number corrected for *Rpl38*. Bars represent means and ranges SD; n = 4. Data are derived from biological replicates. ∗Significantly different between *BGLAP-Cre;Rosa*^*Notch3*^ and controls, *p* < 0.05 by unpaired *t* test.

To explore the direct effect of NOTCH3 in osteocytes, osteocyte-rich preparations from *BGLAP-Cre;Rosa*^*Notch3*^ mice were obtained and analyzed. The expression of *Notch3-NICD* transcripts and the expression of the Notch target genes *Hey1*, *Hey2*, and *HeyL* were increased, whereas mRNA levels for *Sost* and *Dkk1* were suppressed; *Wisp1* mRNA was not changed (Fig. 11). In contrast to the results obtained in intact tibiae and bone marrow stromal cells, there was an induction of *Tnfsf11* (RANKL) but a marked suppression in *Tnfrsf11b* (osteoprotegerin) mRNA levels in osteocytes from *BGLAP-Cre;Rosa*^*Notch3*^ mice (Fig. 11).

### Cocultures of osteoblasts from *Rosa*^*Notch3*^ mice and bone marrow–derived macrophages

To determine the contributions of *Rosa*^*Notch3*^ osteoblasts to osteoclastogenesis, bone marrow–derived macrophages (BMMs) from wildtype mice were cocultured with either bone marrow stromal cells from *BGLAP-Cre;Rosa*^*Notch3*^ or *Dmp1-Cre;Rosa*^*Notch3*^ and control mice or with osteoblasts from *BGLAP-Cre;Rosa*^*Notch3*^ and control mice. Cocultures of bone marrow stromal cells from control or experimental mice with BMMs resulted in the formation of TRAP-positive cells but not in multinucleation, and the cells were limited in number (data not shown). In contrast, multinucleated TRAP-positive cells were induced in BMMs whether they were cultured in the presence of wildtype or *BGLAP-Cre;Rosa*^*Notch3*^ osteoblasts. Moreover, osteoblasts from *BGLAP-Cre;Rosa*^*Notch3*^ mice enhanced osteoclastogenesis from (means ± SD; n = 4) 45 ± 11 osteoclasts in control to 126 ± 19 (*p* < 0.05) osteoclasts in *Rosa*^*Notch3*^ cocultures. The results demonstrate an osteoblast-dependent increase in osteoclast differentiation in cells overexpressing NOTCH3. The effect is congruent with the increased RANKL and decreased osteoprotegerin expression observed in osteocyte-rich cultures from *BGLAP-Cre;Rosa*^*Notch3*^ mice.

## Discussion

In the present study, we explored the consequences of the *Notch3* misexpression in the skeleton. The inactivation of *Notch3* did not result in significant or meaningful changes in bone microarchitecture of either male or female mice studied at a young age or following maturation. These results would indicate that, under basal conditions, NOTCH3, in contrast to NOTCH1 and NOTCH2, is not required for skeletal homeostasis (27, 28, 29). The inactivation of *Notch3* did not cause a decrease in Notch target genes in cells of the osteoblast lineage explaining why neither the *in vitro* cellular nor the *in vivo* skeletal phenotype was altered by the *Notch3* inactivation. Although NOTCH3 regulates RANKL expression in the osteoblast lineage, in accordance with the absence of changes in Notch signaling, the expression of *Tnfsf11* was not affected in either bone marrow stromal cells or osteocytes from *Notch3* null mice (16). There were no changes in gene markers of osteoblast differentiation in bone marrow stromal cells from *Notch3* null mice compared with wildtype cells, although the cells were studied after they had undergone a degree of osteoblast differentiation since they formed mineralized nodules and expressed *Alpl* and *Bglap* (osteocalcin) during the initial phases of the experiment, which was started approximately a week following the isolation of cells. The lack of a skeletal phenotype in *Notch3* null mice may be related to a low level of basal expression or of activation in bone cells or to a possible compensation by the actions of NOTCH1 and NOTCH2. However, this did not require their overexpression since *Notch1*, *Notch2*, and *Notch4* transcript levels were not increased in cells from *Notch3* null mice. The absence of a *Notch3* null phenotype does not mean that NOTCH3 does not play a role in skeletal remodeling under conditions of Notch activation or in the pathogenesis of skeletal disorders associated with a gain-of-NOTCH3 function. Indeed, the expression of the active NOTCH3 NICD in mature osteoblasts and in osteocytes caused a pronounced skeletal phenotype manifested by increased cancellous bone volume and high cortical porosity.

The cancellous bone phenotype observed following the activation of NOTCH3 in osteoblasts and osteocytes can be explained by an induction of osteoprotegerin, leading to an inhibition of bone resorption and decreased remodeling of cancellous bone. The phenotype was suggestive of osteopetrosis since cartilage remnants were found in cancellous bone. An additional mechanism that might have contributed to the phenotype of mice overexpressing NOTCH3-NICD is the activation of Wnt signaling secondary to the suppression of the Wnt antagonists sclerostin and Dkk1 (30). Wnt signaling induces osteoblastogenesis and has a less recognized but important inhibitory effect on osteoclastogenesis and bone resorption secondary to an increase in osteoprotegerin expression by cells of the osteoblastic lineage and to direct effects of Wnt on osteoclast precursors (31, 32, 33, 34, 35). However, the inhibitory effects of NOTCH3 on osteoblast differentiation *in vitro* and bone formation *in vivo* prevailed over the potential stimulatory effects of Wnt. This is in accordance with previous work demonstrating that Notch inhibits osteoblastogenesis and opposes Wnt signaling and Wnt effects in cells of the osteoblast lineage (36, 37, 38).

The effect of NOTCH3-NICD was bone compartment specific, and it caused increased porosity of cortical bone associated with increased intracortical number of osteoclasts and remodeling as demonstrated by an increase in void or pore area. An increase in endocortical surface osteoclasts also was noted. There was a decrease in polar moment of inertia in *Dmp1-Cre;Rosa*^*Notch3*^ male mice indicating impaired bone strength (26). Cortical bone is a tissue rich in osteocytes, and in contrast to the induction of osteoprotegerin in osteoblast cultures, NOTCH3-NICD inhibited osteoprotegerin in osteocyte-rich cell preparations possibly explaining the cortical porosity and enhanced intracortical remodeling. Of interest, RANKL was induced both in bone marrow stromal cells, where its actions could be counteracted by an increase in osteoprotegerin, and in osteocytes where its osteoclastogenic actions prevailed. An additional mechanism responsible for the high cortical porosity could be impaired or incomplete formation of cortical bone or corticalization since both *Bglap* and *Dmp1* are expressed late during embryogenesis and could have induced NOTCH3 (39, 40). Impaired corticalization is suggested by the severity of the cortical bone phenotype and the fact that cortical and trabecular bone were not clearly distinguishable at the distal metaphysis (25). Corticalization is the process by which trabecular bone coalesces at the metaphyseal cortex as longitudinal growth occurs (41, 42). Several genes have been shown to be critical for corticalization, including *Sp7* (Osterix), *Clcn7,* and *Socs3* (25, 41, 43, 44). It is not known whether Notch signaling interacts with any of the genes.

The increase in cancellous bone volume and in cortical porosity following the activation of NOTCH3 are similar to those reported following the supraphysiological activation of NOTCH1 in osteoblasts and osteocytes (29, 45). However, impaired corticalization is not observed in mouse models harboring *Notch2* or *Notch3* mutations that result in modest increments in Notch signaling (16, 17). This would indicate that supraphysiological levels of Notch signaling are necessary to impair corticalization.

Previously, we reported a mouse model (*Notch3*^*em1Ecan*^ or *Notch3*^*tm1.1Ecan*^) of lateral meningocele syndrome presenting with NOTCH3 gain of function due to stabilization of NOTCH3 and osteopenia (16, 46). The bone loss was attributed to increased bone resorption due to an induction of RANKL by cells of the osteoblast lineage. Although the cancellous bone phenotype is in contrast to the one manifested by either the *BGLAP-Cre;Rosa*^*Notch3*^ or the *Dmp1-Cre;Rosa*^*Notch3*^ mouse model described in the present work, the three models of NOTCH3 overexpression presented with cortical bone loss. RANKL is induced in osteoblasts and osteocytes following modest (*Notch3*^*em1Ecan*^) as well as pronounced (*BGLAP-Cre;Rosa*^*Notch3*^ and *Dmp1-Cre;Rosa*^*Notch3*^) levels of NOTCH3 activation, whereas the regulation of osteoprotegerin appears to be dependent on the cell context and degree of Notch activation (45). Indeed, osteoprotegerin is not induced in *Notch3*^*em1Ecan*^ mice explaining their RANKL-dependent resorptive phenotype, is induced in *Rosa*^*Notch3*^ bone marrow stromal cells, possibly explaining the gain in cancellous bone, and is suppressed in *Rosa*^*Notch3*^ osteocytes possibly explaining the cortical bone porosity and osteopenia of mice overexpressing NOTCH3-NICD. Cocultures of bone marrow stromal cells with BMMs did not yield the formation of mature osteoclasts from either control or *Rosa*^*Notch3*^ cells. Cocultures of osteoblasts with BMMs resulted in the formation of mature osteoclasts, and osteoclastogenesis was increased by osteoblasts from *BGLAP-Cre;Rosa*^*Notch3*^ mice. The results are congruent with the increased RANKL and suppressed osteoprotegerin expression observed in osteocytes and could explain the enhanced intracortical remodeling. This is not surprising since the cells are of the same lineage and vary in degree of cell maturation. NOTCH3 overexpression inhibited cancellous bone formation, an effect that could represent direct effects of NOTCH3 on osteoblasts or a degree of decreased bone remodeling in this bone compartment. The phenotype observed in *BGLAP-Cre;Rosa*^*Notch3*^ and *Dmp1-Cre;Rosa*^*Notch3*^ mice was secondary to the exposure of skeletal tissue to supraphysiological levels of Notch activation. This may be relevant to pathological states of Notch signal activation but probably not to a physiological state of bone remodeling.

Because cells of the myeloid/osteoclast lineage do not express *Notch3* mRNA, NOTCH3 is capable of modulating osteoclastogenesis only by indirect mechanisms regulating the expression of RANKL and osteoprotegerin in the osteoblast lineage (16, 47). This is in contrast to NOTCH1 and NOTCH2, which are expressed in the myeloid lineage and have direct, as well as indirect, effects on osteoclastogenesis (14, 17, 18, 48, 49), albeit those of NOTCH1 are inhibitory, whereas those of NOTCH2 are stimulatory.

The present observations may suggest a more important function of NOTCH1 and NOTCH2 than NOTCH3 in skeletal homeostasis since mouse models of gain or loss of function of both receptors exhibit skeletal phenotypes (17, 27, 28, 45). We reported that the dual inactivation of *Notch1* and *Notch2* in *Sp7* (Osterix)-expressing osteoblast precursors or in *Dmp1*-expressing osteocytes increases cancellous bone, and others reported that the inactivation of *Notch2* in osteoblasts causes increased bone mass indicating a negative role of NOTCH1 and NOTCH2 in skeletal homeostasis (27, 28, 29). Similarly, the inactivation of *Notch1* and *Notch2* in the limb bud causes lengthening of the growth plate and an increase in cancellous bone (50).

*Notch1* null mice die during development owing to widespread cellular death, and hypomorphic *Notch2* alleles cause perinatal death due to vascular and renal defects, whereas *Notch3* and *Notch4* null mice develop normally and mutant adults are viable and fertile (51, 52, 53, 54, 55). The present findings are in line with previous work demonstrating that NOTCH3 plays an important function in vascular cells and is required for arterial identity and maturation but it is not essential for bone development (54).

The approach to downregulate Notch signaling has been diverse; however, the approaches are often not specific (56, 57, 58). To target specific Notch receptors, antibodies to the NRR of NOTCH1, NOTCH2, and NOTCH3 have been developed (59, 60). Targeting the NRR prevents cleavage and, therefore, activation of Notch receptors, making it ideal for the specific neutralization of individual Notch isoforms. Previously, we have shown that anti-NOTCH3 NRR antibodies reverse the osteopenia of *Notch3*^*em1Ecan*^ mice. However, anti-NOTCH3 NRR antibodies did not influence the skeletal phenotype of wildtype mice or the behavior of their skeletal cells *in vitro* demonstrating that the prevention of NOTCH3 activation in wildtype cells is without consequences (47). These observations are congruent with the present findings indicating that the basal expression of NOTCH3 is dispensable for skeletal homeostasis.

In conclusion, NOTCH3 activation in osteoblasts/osteocytes increases cancellous bone volume by decreasing bone resorption but causes high cortical porosity by increasing intracortical remodeling. *Notch3* inactivation does not alter bone architecture indicating that basal NOTCH3 activation is dispensable for skeletal homeostasis.

## Experimental procedures

### *Notch3* null mice

*Notch3*^*tm1.1(KOMP)Vlcg*^ mice were created in a C57BL/6N genetic background and were obtained from MMRC; project VG18699 (University of California). *Notch3*^*tm1(KOMP)Vlcg*^ mice in a C57BL/6N genetic background that contained a reporter-tagged null allele were bred at MMRC with Gt(ROSA)26Sortm1(ACTB-cre,-EGFP)Ics/TCP in a C57BL/6N genetic composition to remove the β-actin promoter and the neomycin selection cassette to create *Notch3*^*tm1.1(KOMP)Vlcg*^ mice (61). Frozen sperm from *Notch3*^*tm1.1(KOMP)Vlcg*^ was obtained from MMRC and used for the fertilization of C57BL/6N females at the Center for Mouse Genome Modification at UConn Health. Mice were studied in a C57BL/6N genetic composition following heterozygous intercrosses for the generation of *Notch3*^*−/−*^ and control wildtype sex-matched littermates. Because the yield of the various sex-matched genotypes was modest, litters from various dams had to be pooled so that the study of littermates was not always possible. Genotypes were determined by polymerase chain reaction (PCR) analysis of tail DNA using specific primers (Integrated DNA Technologies; Table 6).

**Table 6:** Primers used for allele identification

Allele	Strand	Sequence	Amplicon size (bp)
Genotyping			
*BGLAP transgene*	Forward	5'-CAAATAGCCCTGGCAGAT-3'	300
	Reverse	5'-TGATACAAGGGACATCTTCC-3'	
*Dmp1 transgene*	Forward	5'-CCCGCAGAACCTGAAGATG-3'	534
	Reverse	5'-GACCCGGCAAAACAGGTAG-3'	
*Notch3*	Forward	5'-GAGGCCCAAGGAATCGAGAC-3'	296
	Reverse	5'-ATGAGACGTTTTCTCCGAGTTCAG-3'	
*Notch3*^*−/−*^	Forward	5'-ACTTGCTTTAAAAAACCTCCCACA-3'	849
	Reverse	5'-CTCCCAAATGTCCCCTGACC-3'	
*Rosa*^*Notch3*^	Forward	5'-CCTCCTGGCTTCTGAGGAC-3'	Wt (Reverse 1) = 333 Floxed (Reverse 2) = 504
	Reverse 1	5'-CTCCGAGGCGGATCACAAGC-3'	
	Reverse 2	5'-CTCGTCCTGCAGTTCATTCA-3'	
*LoxP Recombination*			
*Rosa*^*Notch3*^	Forward	5'-CCTCCTGGCTTCTGAGGAC-3'	Absent *LoxP* recombination (Reverse 1) = 504 Present *LoxP* recombination (Reverse 2) = 432
	Reverse 1	5'-CTCGTCCTGCAGTTCATTCA-3'	
	Reverse 2	5'-ACCTCCCCCATCAGACTCTC-3'	

### *Rosa*^*Notch3*^ mice

*Rosa*^*Notch3*^ mice (Notch3_ICD_YPFTG) were obtained from Dr Spyros Artavanis Tsakonas (Harvard University) and Dr Silvia Fre (Institut Curie) in a C57BL/6 genetic background (20). In these mice, the *Rosa26* locus is targeted with a DNA construct encoding NOTCH3-NICD, preceded by a neo-STOP cassette flanked by *loxP* sites, cloned downstream exon 1 of the *Rosa26* gene. Expression of the NICD from the targeted *Rosa26* locus occurs following the excision of the STOP cassette by Cre recombination of *loxP* sequences. To study the activation of NOTCH3 in osteoblasts and in osteocytes, homozygous *Rosa*^*Notch3*^ mice were mated with either *BGLAP-Cre*^*+/−*^ (Tg(BGLAP-Cre)/Clem/J, Jackson 019509) or *Dmp1-Cre*^*+/−*^ (Tg(Dmp1-Cre)1 Jqfe/Bwd_, Jackson 023047) transgenics to create *Cre*^*+/−*^*;Rosa*^*Notch3*^ experimental and *Rosa*^*Notch3*^ littermate controls (21, 22). Male and female experimental and control mice were compared at 1 month of age. Genotyping of *BGLAP-Cre*, *Dmp1-Cre* transgenics and *Rosa*^*Notch3*^ mice was carried out by PCR in tail DNA extracts (Table 6). Deletion of the *loxP*-flanked STOP cassette by Cre recombinase was documented by PCR in DNA from tibiae using specific primers, and the induction of NOTCH3 NICD and Notch target gene expression in tibiae was documented by quantitative reverse transcription (RT)-PCR in tibiae.

Studies were approved by the Institutional Animal Care and Use Committee of UConn Health.

### Microcomputed tomography

Femoral microarchitecture was determined using a μCT instrument (Scanco μCT 40, Scanco Medical AG), which was calibrated at periodic intervals with a manufacturer provided phantom (62, 63). Femurs from control and experimental mice were scanned in 70% ethanol at high resolution, energy level of 55 peak kilovoltage, intensity of 145 μA, and integration time of 200 ms as reported (16, 17). A total of 100 slices at midshaft and 80 (for *BGLAP-Cre;Rosa*^*Notch3*^ and *Dmp1-Cre;Rosa*^*Notch3*^ and controls) or 160 (for *Notch3*^*−/−*^ and controls) slices at the distal metaphysis were acquired at an isotropic voxel size of 216 μm^3^ and a slice thickness of 6 μm and chosen for analysis. Cancellous bone volume fraction (bone volume/total volume) and microarchitecture were evaluated starting ∼1.0 mm proximal from the femoral condyles. Contours were manually drawn every 10 slices, a few voxels away from the endocortical boundary, to define the region of interest for analysis, whereas the remaining slice contours were iterated automatically. Total volume, bone volume, bone volume fraction, trabecular thickness, trabecular number, connectivity density, structure model index, and material density were measured in trabecular regions using a Gaussian filter (σ = 0.8) and defined thresholds. A threshold of 240 permil equivalent to 355.5 mg of hydroxyapatite (HA)/cm^3^ for 1-month-old and a threshold of 260 permil equivalent to 399.1 mg of HA/cm^3^ for 4-month-old *Notch3*^*−/−*^ and wildtype mice and a threshold of 250 permil equivalent to 377 mg HA/cm^3^ for *BGLAP-Cre;Rosa*^*Notch3*^ and *Dmp1-Cre;Rosa*^*Notch3*^ and control mice were used (62, 63). For analysis of cortical bone, contours were iterated across 100 slices along the cortical shell of the femoral midshaft, excluding the marrow cavity. Analysis of bone volume/total volume, porosity, cortical thickness, total cross-sectional and cortical bone area, segmented bone area, periosteal and endosteal perimeter, and material density, exclusive of cortical pores, and mean polar moment inertia were conducted using a Gaussian filter (σ = 0.8, support = 1) with a threshold of 390 permil equivalent to 682.9 mg of HA/cm^3^ for 1-month-old *Notch3*^*−/−*^, *BGLAP-Cre;Rosa*^*Notch3*^ and *Dmp1-Cre;Rosa*^*Notch3*^ and control mice or a threshold of 400 permil equivalent to 704.7 mg of HA/cm^3^ for 4-month-old *Notch3*^*−/−*^ and control mice.

### Bone histomorphometry

Bone histomorphometry was carried out in 1-month-old mice injected with calcein 20 mg/kg and demeclocycline 50 mg/kg at a 2-day interval and sacrificed 2 days after demeclocycline administration. For cancellous bone analysis, femurs were dissected, fixed in 70% ethanol, and embedded in methyl methacrylate. Femurs were sectioned at a thickness of 5 μm along the sagittal plane on a Microm microtome (Richards-Allan Scientific) and stained with 0.1% toluidine blue. Static and dynamic parameters of bone morphometry were measured in a defined area between 0.35 and 2.16 mm from the growth plate at a magnification of 100x using an OsteoMeasure morphometry system (Osteometrics). Stained sections were used to draw bone tissue and to measure trabecular separation, number and thickness, and osteoid and eroded surface, as well as to count osteoblast and osteoclast number. To assess for the presence of cartilage remnants in cancellous bone, sections were stained with safranin O and fast green. To assess for the presence of TRAP-positive multinucleated cells, bones were decalcified in 14% ethylenediaminetetraacetic acid for 14 days and embedded in paraffin, and 7-μm sections were stained for the presence of TRAP and counterstained with hematoxylin and analyzed at a 100x magnification using OsteoMeasureXP software. Mineralizing surface per bone surface and mineral apposition rate were measured on unstained sections visualized under UV light and a triple diamidino-2-phenylindole/fluorescein/Texas red set long pass filter, and bone formation rate was calculated (64). For cortical histomorphometry, femurs were cut through the mid-diaphysis and a half was frozen and 7-μm sections were cut using a cryostat (Leica CM1950, Leica Biosystems) to assess dynamic parameters of bone histomorphometry. The other half was decalcified and stained for the presence of TRAP as described for cancellous bone, and stained sections were used to count cell numbers and estimate intracortical remodeling.

### Bone marrow stromal cell cultures

Bone marrow stromal cells were obtained from tibiae dissected from 5-week-old *Notch3*^*−/−*^ or *BGLAP-Cre;Rosa*^*Notch3*^ and control mice. Bone marrow cells were isolated by centrifugation following the removal of the epiphyseal ends and recovered in α-minimum essential medium (α-MEM, Life Technologies). Cells were seeded at a density of 1.25 x 10^6^ cells/cm^2^ in α-MEM supplemented with 15% heat-inactivated fetal bovine serum (FBS) and grown in an incubator in an atmosphere of 5% CO_2_ at 37 °C. Cells in suspension were removed by replacing the culture medium 48 h after seeding, and adherent cells were considered bone marrow stromal cells (65). Approximately 5 to 7 days later, adherent cells were digested with trypsin and grown to confluence in α-MEM containing 10% FBS and then switched to α-MEM supplemented with 10% FBS, 100 μg/ml ascorbic acid, and 5 mM β-glycerophosphate (all from Sigma-Aldrich) (66, 67, 68). Culture plates were stained with Alizarin red for visualization of mineralized nodules, or cell extracts were obtained for RNA determinations.

### Osteocyte-enriched cell preparations

Osteocyte-enriched cells were obtained from 1-month-old *Notch3*^*−/−*^ or *BGLAP-Cre;Rosa*^*Notch3*^ and control mice following a modification of a previously described method (48, 69). Tibiae were removed aseptically from 1-month-old experimental and control mice. Tibiae were dissected free from surrounding tissues, the proximal epiphyseal end was excised, and the bone marrow was removed by centrifugation. The distal epiphysis was excised, and bones were digested for 20 min at 37 °C with type II bacterial collagenase pretreated with *N*-α-tosyl-l-lysyl-chloromethyl ketone hydrochloride and subsequently exposed to 5 mM EDTA for 20 min at 37 °C. The resulting osteocyte-enriched cortical bones were extracted for RNA determinations or cultured overnight in Dulbecco's modified Eagle's medium supplemented with 10% FBS prior to RNA extraction (48, 70).

### Osteoblast-enriched cells and cocultures with bone marrow–derived macrophages

To obtain osteoblast-enriched cells, parietal bones from 3- to 5-day-old *BGLAP-Cre;Rosa*^*Notch3*^ and littermate wildtype mice were exposed to 1.2 units/ml Liberase TL (Sigma-Aldrich) for 20 min at 37 °C, and cells were extracted in five consecutive reactions (48, 71). Cells from the last three digestions were pooled and seeded at a density of 10,000 cells/cm^2^ and cultured in Dulbecco’s modified Eagle’s medium supplemented with nonessential amino acids (both from Thermo Fisher Scientific), 20 mM Hepes, 100 μg/ml ascorbic acid (both from Sigma-Aldrich), and 10% heat-inactivated fetal bovine serum (Atlanta Biologicals) in a humidified 5% CO_2_ incubator at 37 °C. BMMs from wildtype mice were isolated by flushing of the marrow as described (48, 49). Cells were centrifuged, and the sediment was suspended in α-MEM (Thermo Fischer Scientific) in the presence of 10% FBS and recombinant human macrophage colony stimulating factor at 30 ng/ml as described (48, 49). To determine the contribution of osteoblast-derived factors to osteoclast formation, bone marrow stromal cells or calvarial osteoblast-enriched cells from *BGLAP-Cre;Rosa*^*Notch3*^ and wildtype control littermates were seeded at a density of 15,700 cells/cm^2^ in α-MEM in the presence of BMMs from wildtype mice at a density of 47,000 cells/cm^2^ and cultured in the presence of 1,25-dihydroxyvitamin D_3_ at 10 nM (16). Cultures were carried out until the formation of multinucleated TRAP-positive cells was achieved. TRAP enzyme histochemistry was conducted using a commercial kit (Sigma-Aldrich), in accordance with the manufacturer’s instructions, as reported (48). TRAP-positive cells containing three or more nuclei were considered osteoclasts.

### Quantitative reverse transcription–polymerase chain reaction

Total RNA was extracted from cells with the RNeasy kit (Qiagen) and from homogenized tibiae or osteocyte-enriched preparations with the micro RNeasy kit (Qiagen), in accordance with manufacturer's instructions, as reported (17, 48, 72, 73). The integrity of the RNA from tibiae and osteocyte-rich fragments was assessed by microfluidic electrophoresis on an Experion instrument (Bio-Rad), and only RNA with a quality indicator number equal to or higher than 7.0 was used for subsequent analysis. Equal amounts of RNA were reverse transcribed using the iScript RT-PCR kit (Bio-Rad) and amplified in the presence of specific primers (Integrated DNA Technologies) (Table 7) with SsoAdvanced Universal SYBR Green Supermix (Bio-Rad) at 60 °C for 35 cycles. Transcript copy number was estimated by comparison with a serial dilution of cDNA for *Alpl* (encoding for alkaline phosphatase from American Type Tissue Culture Collection (ATCC), *Bglap* (encoding for osteocalcin; from J. Lian, University of Vermont), *Dkk1* (from Thermo Fisher Scientific), *Hes1* (from ATCC), *Hey1* and *Hey2* (both from T. Iso, Gunma University), *HeyL* (from D. Srivastava, Gladstone Institute of Cardiovascular Disease or Dharmacon), *Notch2* and *Sost* (from Thermo Fisher Scientific), *Notch1* (from J.S. Nye), *Notch4* (from Y. Shirayoshi), *Tnfsf11* (encoding for RANKL from Source BioScience), *Tnfrsf11b* (encoding for osteoprotegerin from ATCC), or *Wisp1* (from ATCC) (74, 75, 76, 77, 78). *Notch3* copy number was estimated by comparison with a serial dilution of ∼100–base pair synthetic DNA template (Integrated DNA Technologies) cloned into pcDNA3 as described, and Notch3 NICD was estimated by comparison with a CMV/N31CHA plasmid (Addgene 47618) (15, 79, 80). Amplification reactions were conducted in a CFX96 qRT-PCR detection system (Bio-Rad), and fluorescence was monitored during every PCR cycle at the annealing step. Data are expressed as copy number corrected for *Rpl38* (from ATCC) (81).

**Table 7:** Primers used for qRT-PCR

Gene	Strand	Sequence	GenBank accession number
*Alpl*	Forward	5'-TGGTATGGGCGTCTCCACAGTAACC-3'	NM_007431
	Reverse	5'-CTTGGAGAGGGCCACAAAGG-3'	
*Bglap*	Forward	5'-GACTCCGGCGCTACCTTGGGTAAG-3'	NM_001037939
	Reverse	5'-CCCAGCACAACTCCTCCCTA-3'	
*Dkk1*	Forward	5'-CCCTCCCTTGCGCTGAAGATGAGGAGT-3'	NM_010051
	Reverse	5'-CGCTTTCGGCAAGCCAGAC-3'	
*Hes1*	Forward	5′-ACCAAAGACGGCCTCTGAGCACAGAAAGT-3′	NM_008235
	Reverse	5′-ATTCTTGCCCTTCGCCTCTT-3′	
*Hey1*	Forward	5′-ATCTCAACAACTACGCATCCCAGC-3′	NM_010423
	Reverse	5′-GTGTGGGTGATGTCCGAAGG-3′	
*Hey2*	Forward	5′-AGCGAGAACAATTACCCTGGGCAC-3′	NM_013904
	Reverse	5′-GGTAGTTGTCGGTGAATTGGACCT-3′	
*HeyL*	Forward	5′-CAGTAGCCTTTCTGAATTGCGAC-3′	NM_013905
	Reverse	5′-AGCTTGGAGGAGCCCTGTTTC-3′	
*HeyL*^a^	Forward	5'-TCCTCACCCGTCAGA-3′	NM_013905
	Reverse	5'-AGGCACCATGTAACTCA-3′	
*Notch1*	Forward	5'-GTCCCACCCATGACCACTACCCAGTTC-3′	NM_008714
	Reverse	5'-GGGTGTTGTCCACAGGGGA-3′	
*Notch2*	Forward	5'-TGACGTTGATGAGTGTATCTCCAAGCC-3′	NM_010928
	Reverse	5'-GTAGCTGCCCTGAGTGTTGTGG-3′	
*Notch3*	Forward	5′-CCGATTCTCCTGTCGTTGTCTCC-3′	NM_008716
	Reverse	5′-TGAACACAGGGCCTGCTGAC-3′	
*Notch3NICD*	Forward	5′-CATCCTTATTTGACCCCGTC-3′	NM_008716
	Reverse	5′-TGGCATTGGTAGCAGTTG-3′	
*Notch4*	Forward	5′-CCAGCAGACAGACTACGGTGGAC-3′	NM_010929
	Reverse	5′-GCAGCCAGCATCAAAGGTGT-3′	
*Rpl38*	Forward	5′-AGAACAAGGATAATGTGAAGTTCAAGGTTC-3′	NM_001048057; NM_001048058; NM_023372
	Reverse	5′-CTGCTTCAGCTTCTCTGCCTTT-3′	
*Sost*	Forward	5′-AGGAATGATGCCACAGAGGTC-3′	NM_024449
	Reverse	5′-CTGGTTGTTCTCAGGAGGAGGCTC-3′	
*Tnfrsf11b*	Forward	5′-CAGAAAGGAAATGCAACACATGACAAC-3′	NM_008764
	Reverse	5′-GCCTCTTCACACAGGGTGACATC-3′	
*Tnfsf11*	Forward	5′-TATAGAATCCTGAGACTCCATGAAAAC-3′	NM_011613
	Reverse	5′-CCCTGAAAGGCTTGTTTCATCC-3′	
*Wisp1*	Forward	5′-TCCAGGAGTTAAGTGATTTGCTCA-3′	NM_018865
	Reverse	5′-CATGTTACATGACACTGGGCTTC-3′	

GenBank accession numbers identify transcript recognized by primer pairs.

a Used for experiment shown in Figure 3.

### Immunoblotting

Bone marrow stromal cells from control or *BGLAP-Cre;Rosa*^*Notch3*^ mice were extracted in buffer containing 25 mm Tris-HCl (pH 7.5), 150 mm NaCl, 5% glycerol, 1 mm EDTA, 0.5% Triton X-100, 1 mm phenylmethylsulfonyl fluoride, and a protease inhibitor mixture (all from Sigma-Aldrich). Quantified total cell lysates (35 μg of total protein) were separated by sodium dodecyl sulfate polyacrylamide gel electrophoresis in 8% or 10% polyacrylamide gels and transferred to Immobilon-P membranes (Millipore). The blots were probed with anti-NOTCH3 (ab23426) (Abcam) and anti-β-Actin (3700) antibodies (Cell Signaling Technology) and exposed to anti-rabbit or anti-mouse IgG conjugated to horseradish peroxidase (Sigma-Aldrich) and incubated with a chemiluminescence detection reagent (Bio-Rad). Chemiluminescence was detected on a ChemiDoc XSR+ molecular imager (Bio-Rad) with Image Lab software (version 6.0.1), and the amount of protein in individual bands was quantified (49).

### Statistics

Data are expressed as individual sample values, and means ± SD. All data represent biological replicates except for stromal cell cultures shown in Figure 10 and BMM/osteoblast coculture experiments, which represent technical replicates. Quantitative RT-PCR (qRT-PCR) values were derived from two technical replicates of biological replicates as indicated in the text and legends. Statistical differences were determined by unpaired Student's *t* test for pairwise comparisons or two-way analysis of variance for multiple comparisons with Holm–Šidák post hoc analysis.

## Data availability

Data not shown will be shared upon request to Ernesto Canalis at canalis@uchc.edu.

## Conflict of interest

The authors declare that they have no conflicts of interest with the contents of this article. Dr Zanotti is currently employed by Dyne Therapeutics.
